# Exploring the host-pathogen interaction and genome analysis of multidrug-resistant bacterial pathogen *Proteus penneri* isolated from *Labeo rohita*

**DOI:** 10.3389/fimmu.2026.1733414

**Published:** 2026-03-13

**Authors:** Vikash Kumar, Basanta Kumar Das, Suvra Roy, Pratyasha Bhowal, Arpita Roy, Timothy J. Bruce, Jorge Galindo-Villegas

**Affiliations:** 1 Aquatic Environmental Biotechnology (AEB) Division, Indian Council of Agricultural Research (ICAR)-Central Inland Fisheries Research Institute (CIFRI), Barrackpore, India; 2 Department of Biotechnology and Dr. Biresh Chandra Guha Centre for Genetic Engineering and Biotechnology, University of Calcutta, Kolkata, West Bengal, India; 3 School of Fisheries, Aquaculture and Aquatic Sciences, Auburn University, Auburn, AL, United States; 4 Department of Genomics, Faculty of Biosciences and Aquaculture, Nord University, Bodø, Norway

**Keywords:** antimicrobial resistance, aquaculture, disease, *myd88*, pathogenesis

## Abstract

Multidrug-resistant (MDR) bacterial pathogens represent an escalating challenge to sustainable aquaculture, particularly in high-value freshwater species such as *Labeo rohita*, a cornerstone of South Asian aquaculture. This study provides the first comprehensive integration of genomic, immunological, and microbiome analyses to characterize *Proteus penneri* as an emerging MDR pathogen associated with severe disease manifestations in *L. rohita*, including exophthalmia, ulceration, and hemorrhage. Robust identification through biochemical assays, 16S rRNA sequencing, and phylogenetic analysis confirms the clinical relevance of this isolate. Functional assays demonstrated pronounced virulence, evidenced by hemolysin activity, extensive histopathological damage, and dose-dependent mortality, underscoring its pathogenic capacity *in vivo*. The observed resistance to multiple frontline antibiotic classes, including tetracyclines, macrolides, and carbapenems, highlights a critical therapeutic limitation in aquaculture settings. Genomic analysis further revealed a diverse repertoire of antimicrobial resistance genes, virulence determinants (notably biofilm formation and secretion systems), and mobile genetic elements, suggesting a strong potential for persistence, adaptability, and horizontal gene transfer. Infection-associated gut microbiome disruption, marked by elevated MAR indices and enrichment of virulence-associated taxa, indicates that *P. penneri* not only exploits host tissues but also reshapes the microbial ecosystem in ways that may exacerbate disease severity and resistance dissemination. Concurrently, heightened serum cortisol, *C3*, and *Hsp70* levels, along with transcriptional upregulation of key immune and stress-related genes (*hsp70, nod, il6, sod, c3*, and *myd88*), reflect an intense pro-inflammatory and physiological stress response. In silico docking analyses implicating *myd88*–lipopolysaccharide interactions provide mechanistic insight into potential immune-modulatory strategies employed by the pathogen. Collectively, these findings delineate a multifactorial basis for *P. penneri* virulence and MDR, emphasizing its significance as an emerging aquaculture pathogen. Future research should prioritize functional validation of key virulence and resistance genes, longitudinal surveillance to assess transmission dynamics and AMR spread, and experimental evaluation of alternative disease mitigation strategies, including probiotics, phage therapy, and immune-modulating interventions, to reduce antibiotic reliance and enhance fish health resilience in aquaculture systems.

## Introduction

Aquaculture plays a vital role in the global food supply, aiding in feeding millions and promoting sustainable food security. It provides more than half of the fish consumed by humans and sustains millions of livelihoods, especially in low- and middle-income countries (1). In South Asia, *Labeo rohita*, one of the Indian major carps, holds substantial nutritional and economic importance (2). However, the sustainability of aquaculture is increasingly threatened by bacterial diseases, which result in high mortality rates, reduced productivity, and increased reliance on antibiotics. This overreliance accelerates the emergence of multidrug-resistant (MDR) pathogens (3). MDR pathogens compromise therapeutic efficacy and exacerbate environmental dissemination of antimicrobial resistance (AMR) genes, with consequences for both ecosystem and human health (4).

Among emerging pathogens, *Proteus penneri* is gaining recognition in aquaculture. This Gram-negative bacillus, widely distributed in aquatic and terrestrial habitats, is known to cause opportunistic infections in humans and animals (5). Recent evidence has associated *P. penneri* with severe disease outbreaks and high mortality in cultured fish, including *L. rohita* (6). Unlike well-characterized fish pathogens such as *Aeromonas* or *Vibrio*, *P. penneri* lacks comprehensive genomic and immunological profiling. Its MDR profile and virulence factors, such as biofilms and secretion systems, are poorly characterized, but it is inferred that they contribute to disease severity. The molecular mechanisms underlying its pathogenicity and host interactions remain poorly understood (7). The intensification of aquaculture practices, combined with climate-driven stressors, has heightened the risk of bacterial disease emergence and progression (8). Shifts in environmental conditions can alter host-pathogen dynamics, compromise fish immune responses, and promote the emergence of novel bacterial strains. Therefore, improved understanding of disease mechanisms and host-pathogen interactions is critical for developing effective control strategies and achieving resilience in aquaculture systems.

In 2022, a severe hemorrhagic disease outbreak occurred in the *L. rohita* farm in West Bengal, India, resulting in mortality exceeding 60%. Hence, the objective of our study was to comprehensively characterize the etiological agent responsible for the outbreak and to elucidate its pathogenic mechanisms, antimicrobial resistance potential, and host-pathogen interactions in *L. rohita*. In response, the causative agent was isolated from moribund fish and identified as *P. penneri* based on morphological, biochemical, and 16S rDNA analyses. To investigate its pathogenic potential, an isolate was subjected to whole genome sequencing, antimicrobial resistance profiling, and virulence assessments, including hemolysin activity and histopathology. Experimental infection models were employed to confirm its ability to induce disease under controlled conditions. Beyond pathogen profiling, this study also investigated host responses, including shifts in gut microbiota, changes in serum biochemical parameters, and the expression of immune-related genes. *In silico* docking analysis was additionally performed to evaluate potential interactions between host immune components and *P. penneri*-derived virulence factors. The comprehensive dataset presented here contributes novel insights into *P. penneri* pathogenesis and host response, establishing a foundation for targeted disease management and future research in freshwater aquaculture.

## Materials and methods

### Outbreak description

**Figure 1:**
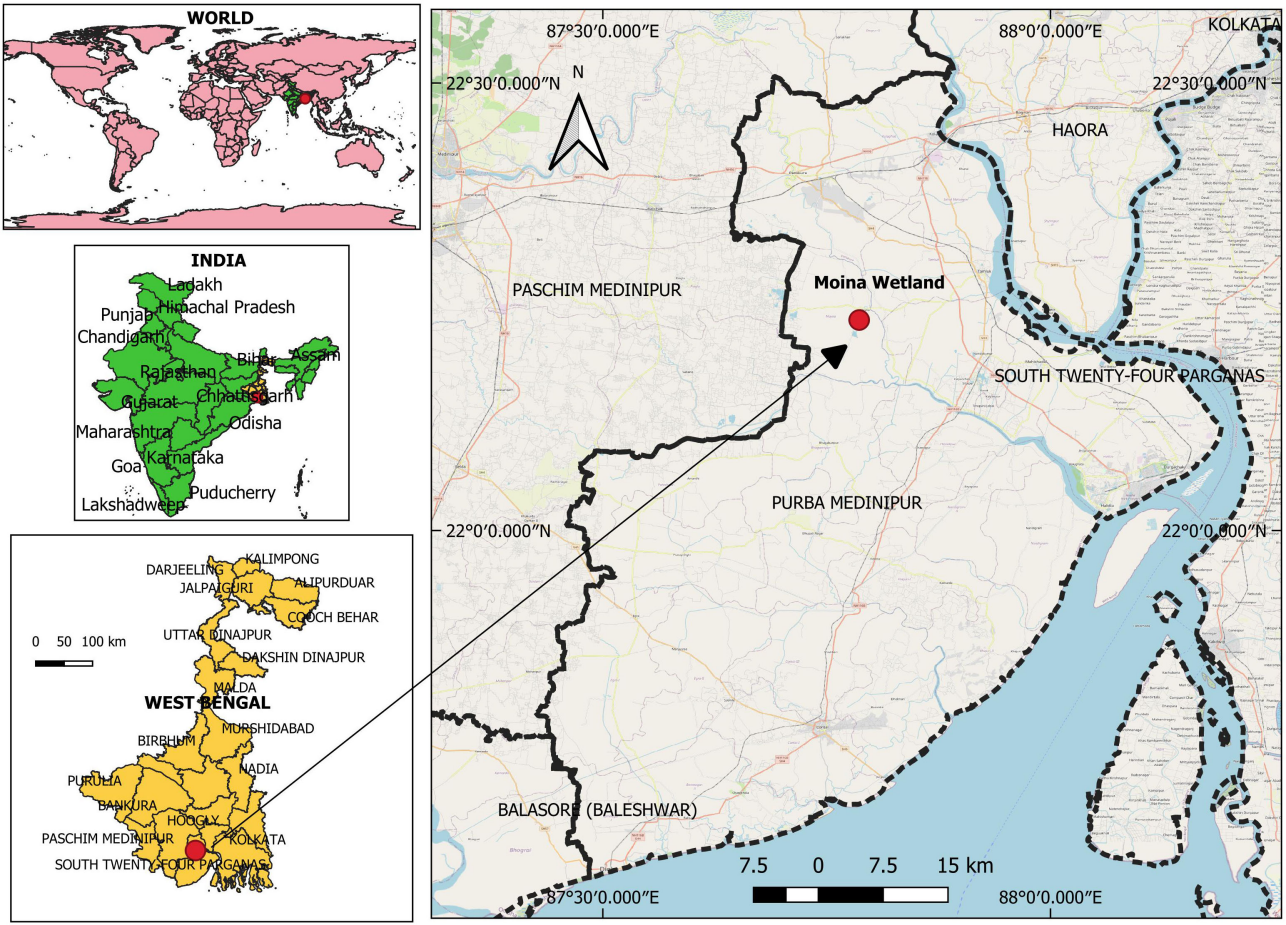
The aquaculture site in Moina, West Bengal, India, reported mass mortality of *Labeo rohita*.

Between mid-July and early August 2022, a severe mortality incident was investigated in *L. rohita* juveniles (length: 140.4 ± 8.1 mm, weight: 15.32 ± 2.1 g) at aquaculture farms (22°18’33 “N; 87°50’20” E) in Moina, Purba Medinipur district of West Bengal, India (**Figure 1**). The farms were raising a mix of fish species, including *L. rohita*, *L. catla*, *Cirrhinus mrigala*, and *Hypopthalmichthys molitrix*. A team of researchers from ICAR-CIFRI, Kolkata, India, visited the facility as the central nodal agency for fish disease surveillance. During our visit, we estimated a mortality rate of around 55% in *L. rohita* based on passive data collection. The fish displayed signs of disease, including extreme lethargy, hemorrhage, ulceration, discoloration, and redness on their fins and bodies. A total of 30 symptomatic moribund rohu exhibiting clinical signs were collected from two aquaculture farms. Similarly, 30 asymptomatic fish from nearby aquaculture ponds were also collected and transferred to ICAR-CIFRI, Kolkata Fish Pathology Lab, for screening for etiological agents. Fresh smears of skin and gills were examined under a microscope in the lab to check for ectoparasites. The fish were then euthanized by an overdose of MS222 (150 mg/L for ~2 min) (Sigma-Aldrich), and post-mortem exams were carried out to document various clinical signs in the internal organs. For molecular studies, different tissues (liver) from symptomatic and asymptomatic *L. rohita* were collected in RNA later. Further, the gut tissue samples were fixed in neutral buffered formalin (10%) for histopathological analysis (details summarized in the graphical abstract prepared in Canva software).

### Bacterial isolation from moribund fish

Fish exhibiting distinct clinical signs were anesthetized with clove oil (Merck, Germany) at a dosage of 50 μL/L. The tissue samples, including liver, kidney, and blood samples, were collected aseptically under sterile conditions. The tissues were homogenized in phosphate-buffered saline (PBS), and 200 μl aliquots were inoculated onto TSA (tryptic soy agar) (Himedia, India) and incubated for 24 hours at 28°C. The emergence of morphologically uniform colonies indicated the presence of a single bacterial strain. A representative colony was subcultured onto fresh TSA plates and incubated under identical conditions to obtain a pure culture. The isolate was then transferred to tryptic soy broth (TSB) and incubated for 24 hours at 28°C. A glycerol stock was prepared (30%) and stored at -20°C for future use. Furthermore, before downstream applications such as hemolysin assays, biochemical characterization, antibiotic susceptibility testing, challenge trials, and DNA extraction, the bacterial strain was enriched in APW (alkaline peptone water) to facilitate optimal growth (9, 10).

### Bacteria identification by 16S rRNA gene and phylogenetic analysis

The standard Sarkosyl method was employed to isolate genomic DNA from the bacteria based on chemical cell lysis and removal of proteins and other cellular components (11). In this method, bacterial cells are first harvested and suspended in an appropriate buffer, often containing Tris and EDTA, where EDTA chelates divalent cations and helps inhibit DNase activity. Sarkosyl (N-lauroyl sarcosine), an anionic detergent, is then added to disrupt the bacterial cell membrane and solubilize lipids and membrane proteins, thereby facilitating efficient cell lysis and the release of genomic DNA. Proteinase K is frequently included to digest cellular proteins, including nucleases and histone-like proteins, ensuring the DNA remains intact. Following lysis, contaminants such as proteins and cell debris are removed by phenol–chloroform extraction or salt precipitation, and the DNA is subsequently precipitated using ethanol or isopropanol. The resulting genomic DNA is then washed, dried, and resuspended in buffer or sterile water, yielding high-molecular-weight DNA suitable for downstream molecular biology applications (12).

The quality of the DNA was assessed on a 1% agarose gel and quantified using a NanoDrop. The 16S rRNA gene was amplified using the GeneAmp PCR System 9700 with universal bacterial primers. A 50 μL PCR mixture included 10× buffer, MgCl2, dNTPs, primers, genomic DNA, and Taq polymerase. PCR involved an initial denaturation step at 95°C for 2 min, followed by 35 cycles of denaturation at 94°C for 30 seconds, annealing at 52°C for 60 seconds, and extension at 72°C for 90 seconds, with a final extension at 72°C for 7 minutes. PCR products were visualized on a 1.8% agarose gel. (13). The amplified gene fragments were sequenced in both directions using an ABI 373xl capillary sequencer (Applied Biosystems, Foster City, CA, USA). Using DNA Baser 7.0.0, the forward and reverse sequences were aligned to generate a contig. The sequence data were submitted to GenBank (accession number OP554277), and a phylogenetic tree was constructed using MEGA X via the Neighbor-Joining method.

### Biochemical characterization of isolated bacteria

The biochemical characterization of bacterial isolates was performed using Bergey’s Manual of Systematic Bacteriology (14). The isolated bacteria were later identified by their cell wall characteristics using the Gram stain method. Later, it was analyzed for biochemical activity through various tests such as ONPG (for β-galactosidase), lysine and ornithine utilization, urease activity, melibiose, phenylalanine deamination, nitrate reduction, hydrogen sulfide production, citrate metabolism, esculin hydrolysis, rhamnose, Voges Proskauer’s (VP), raffinose, methyl red, indole production, malonate utilization, and the fermentation of different carbohydrates like arabinose, xylose, adonitol, cellobiose, saccharose, trehalose, glucose, and lactose. The assessment also included oxidase activity (KB003, Hi-media).

### Antibiogram assay

Antimicrobial susceptibility testing was performed using the standard disk diffusion method on Mueller–Hinton agar (15). The isolate was cultured in sterile Mueller–Hinton broth at 28°C for 24 h and adjusted to approximately 10⁶ CFU/mL prior to plating. Commercially available antibiotic discs (HiMedia, India) representing multiple antimicrobial classes were applied to the inoculated agar plates under sterile conditions. Plates were incubated at 28°C for 24 h, and inhibition zone diameters were measured in millimeters. Susceptibility was categorized as sensitive (S), intermediate (I), or resistant (R) according to Clinical and Laboratory Standards Institute (CLSI) guidelines (16, 17). The multiple antibiotic resistance (MAR) index was calculated as previously described (18, 19). All assays were conducted in independent replicates to ensure reproducibility.

### Whole genome sequencing of a pathogenic *P. penneri* isolate

#### Sequencing, assembly, and genome annotation

The entire genome of the *P. penneri* isolate was assembled and annotated using the PacBio HiFi reads system and P6-C4 chemistry on a single-molecule real-time (SMRT) cell per genome. The PacBio Sequel II-generated raw subreads were converted into HiFi reads, which were used as input for genome assembly in the subsequent step. The genome was assembled using Canu *de novo* assembler, an essential tool for assembling intricate genomes from long-read sequencing data, and has gained widespread acceptance in the genomics community due to its robustness, accuracy, and capacity to manage repetitive and difficult regions within genomes (20). To determine their genome completeness, the tools “QUAST” (21) and “BUSCO” (22) were utilized. The genomic characteristics were done using a subsystems technology tool kit (RASTtk) in BV-BRC (23).

#### Genomic analysis and bioinformatics

Multiple genomic analysis tools were used in this study, which were performed on the Bacterial and Viral Bioinformatics Resource Center (BV-BRC) server. The Comprehensive Genome Analysis was conducted using the BV-BRC server’s meta-service, which analyzes raw data and single or paired reads to determine genome assembly, annotation, quality control, AMR, and specific genes with key functions. Genome annotation involves identifying the genome sequence along with functional components. The BV-BRC server used rapid annotation with a subsystems technology toolkit (RASTtk) to annotate genomic features. ProkSee was used for alternative genome visualization and annotation (24). Investigating phylogenetic relationships in bacterial populations is essential for understanding the molecular evolutionary history (25). To assess the evolutionary relationships between our strains and other outgroup strains, we employed the BV-BRC phylogenetic tree known as “Codon Trees.” This method relies on predefined protein global families, such as PATRIC (PGFams) (26), which select 10–1000 single-copy families from genomic group members. Muscle was employed to generate alignments for protein sequences across all families (27), while nucleotide sequences were aligned using BioPython’s codon-align function (28). To calculate confidence values, bootstrap rounds of 100 rapid were executed within RaxML (29). The genome was further compared using the TYGS (Type Strain Genome Server) for taxonomic relatedness (30). Functional annotation was done using KEGG Mapper (31).

### Experimental design for *in vivo* survival assay

Healthy fingerlings of *L. rohita* (mean length = 125.1 ± 1.9 mm; mean weight = 14.4 ± 2.8 g) were obtained from a nearby fish hatchery. All fish (n = 250) showed normal behavior and morphology, with no visible signs of illness, including hemorrhages, discoloration, ulcers, scale loss, or surface redness. Before the experiment, the fish were randomly selected and screened for microbes using standard bacterial culture methods (swabs were collected from fish, cultured in growth medium, and virulence was assessed using a survival assay) (32, 33). Later, for two weeks, the fish (n = 250) were acclimatized in 200 L Fiber-reinforced plastic (FRP) tanks under controlled conditions. The fish were fed with a floating commercial feed (30% crude protein, 5% crude lipid) at a rate of 3-5% of their body weight, administered twice daily. The culture water in the control group was exchanged daily with 60% with freshwater. Dissolved oxygen, temperature, pH, and salinity were measured *in situ* every two days using a portable multiparameter photometer (Hanna Instruments, Belgium), a pH meter (VWR, Belgium), and a refractometer (VWR, Belgium). Dissolved inorganic nitrogen, i.e., nitrite nitrogen (NO_2_--N) and nitrate nitrogen (NO_3_--N), was determined every two days using a multiparameter photometer (Hanna Instruments, Belgium) according to the manufacturer’s instructions. The physicochemical parameters of water were maintained in accordance with the standard protocol of APHA (34). All experiments were performed according to the animal utilization protocol approved by the Institutional Animal Ethics Committee, ICAR-CIFRI, Kolkata, India (CIFRI-IAEC/17/2023-24) for the experimental setup. All procedures were carried out with maximal effort to minimize fish suffering.

### Bacteria culture and survival assay

For the experiment, 20 mL of sterile TSB (Tryptone Soya Broth) was used in a 50 mL Erlenmeyer flask (Himedia, India) to culture the bacterial strain (*P. penneri*) for 24 hours at 28°C. Subsequently, after centrifugation at 5,000 rpm for 5 minutes, the bacterial cells were harvested and subjected to three washes with a sterile saline solution. The resulting pellets were resuspended in saline, and the cell concentration was determined using the spread plate technique (CFU/mL). Twenty fish per concentration were intraperitoneally injected with 200 μL of bacterial suspension containing 1.2 × 10¹ to 1.2 × 10^7^ CFU/mL. Control fish received 200 µL of saline solution. The fish were subsequently maintained in an FRP tank and monitored at 24-hour intervals for 120 hours (35, 36). During the assay period, dissolved oxygen, temperature, pH, and salinity, as well as dissolved inorganic nitrogen (i.e., nitrite nitrogen (NO_2_-N) and nitrate nitrogen (NO_3_-N), were determined every two days using a multiparameter photometer according to the manufacturer’s instructions (**Supplementary Table S1**). To verify Koch’s postulate, the bacteria were re-isolated and identified from the liver, kidney, and blood of the moribund fish. The assay was performed in quintuplicate and is representative of two independent experiments.

### Hemolysin assay

The hemolytic activity of the isolated bacterial strain was determined using a standard protocol with some minor changes (37). In brief, we prepared TSA (Tryptone Soya Agar, India) plates supplemented with defibrinated sheep blood (5%). Then, we grew stock pure cultures of the bacterial strain in TSB overnight at 28°C with gentle agitation. Next, we diluted the overnight culture to an optical density (OD) of 0.5 at 600 nm and carefully spotted 2 µl of this diluted culture onto the center of the hemolysin test plates. We incubated the plates for 48 hours at 28°C. After incubation, the diameter of the hemolytic zone was measured using calipers (Anyi Instrument, Guilin, China) to determine the extent of hemolysis. To ensure consistency, we repeated the entire process five times, using fresh media for each run.

### Cellular ultrastructure analysis (histology)

Fish from both control and treatment groups (n = 5 per group) were anesthetized through Clove oil (50 µL/L) at 48 and 96 h post-experiment for gut tissue collection. The post-mortem analysis was conducted to document any gross lesions or pathological signs in the internal organs. Gut tissue samples were excised and immediately fixed in 10% neutral buffered formalin (NBF). Fixed tissues were washed, trimmed into 1–2 mm segments, and then subjected to a graded ethanol dehydration series, followed by clearing in xylene. The cleared tissue was embedded in paraffin using an impregnation technique (Leica EG 1140H, Germany). Paraffin-embedded blocks were sectioned at a thickness of five μm using a rotary microtome, followed by staining with hematoxylin and eosin (38). Histological sections were examined through a light microscope to evaluate cellular changes.

### Role of *P. penneri* on gut microbiota and immunity

#### Bacterial challenge assay

At first, the *P. penneri* dose-inducing mortality in *L. rohita* fingerlings, near 80% in 168 h (from the previous experiment, i.e., 1.2 × 10^6^), was considered as an optimum infectious dose for the challenge assay to evaluate the *P. penneri*-induced effect on gut microbiota, immunity, and survival of the host. A group of *L. rohita* fingerlings was injected intraperitoneally (20 numbers) with 200 µL of 1.2 × 10^6^ bacterial suspension. The fish in the control group were injected with saline solution (200 µL). Subsequently, gut samples were collected for bacterial enumeration, serum samples for biochemical analysis, and liver tissue samples for gene expression analysis.

### Bacterial enumeration from gut samples of *L. rohita*

The gut samples of *L. rohita* were collected to determine the total cultivable bacteria abundance, following the established protocol by Guan et al. (39) with minor changes (39). In brief, the fish were randomly selected from control groups at 24, 72, and 120 hours post-treatment, placed in sterile plastic bags at 4°C, and processed within 24 hours of collection. After disinfecting the *L. rohita* surface with 70% alcohol, we dissected it in a sterile environment. We then removed the intestines, cut them into small pieces, and homogenized the tissue samples in 10 mL of distilled water for 15 to 30 seconds at room temperature, using sterile techniques. At the same time, the homogenates were serially diluted using sterile physiological saline, and 0.1 mL aliquots from each dilution were spread onto Petri dishes with TSA (Tryptone Soya Agar, India) media. These plates were incubated overnight with shaking at 120 rpm and 28°C. Colonies within the range of 30 to 300 CFU/mL at specific dilutions were counted to determine the bacterial abundance in the fish gut samples.

### Characterization of bacterial isolates enumerated from gut samples of *L. rohita*

#### Molecular identification by 16S rRNA gene and phylogenetic comparison of bacterial isolates

The isolated bacterial strains were identified using PCR 16S rRNA amplicon sequencing, a process detailed in the **Supplementary Methods** and **Supplementary Table S1**, which includes isolate preparation for PCR, DNA extraction, and primer details. The PCR products were then sequenced in both directions using the reliable ABI 373xl capillary sequencer (Applied Biosystems, Foster City, CA). To assemble the 16S rRNA sequences, forward and reverse reads were carefully aligned using DNA Baser 7.0.0. These sequences were then compared with existing ones in the NCBI GenBank via BLAST (http://blast.ncbi.nlm.nih.gov). Finally, the sequences were submitted to GenBank to aid in creating a phylogenetic tree, thereby advancing our understanding of these bacteria.

We carried out evolutionary analyses using MEGA11 with the Neighbor-Joining method to map out the evolutionary history of the bacterial strains we recovered, resulting in an optimal tree (40, 41). This tree is drawn to scale, meaning that its branch lengths correspond to the same units as the evolutionary distances we used to build it. We calculated these distances using the Maximum Composite Likelihood method, and the units reflect the number of base substitutions per site (42). Our analysis included 32 nucleotide sequences, considering codon positions 1, 2, and 3, as well as non-coding regions. Ambiguous positions were carefully removed between each sequence pair using the pairwise deletion option, resulting in a final dataset of 1444 positions.

### Virulence of isolated bacterial strains

#### Survival assay

The isolated bacterial strains were subcultured separately in sterile Tryptone Soya Broth (TSB) in 20 mL within 50 mL of Erlenmeyer flasks (Himedia, India) at 28°C for 24 hours. The bacterial cells were gently collected by centrifugation at 5000 rpm for 5 minutes. Afterward, they were carefully washed three times with sterile saline solution. The resulting pellets were then resuspended in sterile saline solution, and the bacterial concentration was estimated using the spread plate method to ensure an accurate measurement. Intraperitoneal injections of the experimental *L. rohita* (20 fish per concentration) were administered with bacterial suspension with 1.2 × 10^7^ CFU/ml. Control fish received an injection of 0.2 mL of sterile saline solution. Following this, the fish were maintained in an FRP tank and monitored every 24 hours over a total duration of 168 hours. To validate Koch’s postulate, bacteria were re-isolated and identified from the kidney, liver, and blood samples of moribund fish. Similarly, as mentioned above, the assay was performed in quintuplicate and is representative of two independent experiments (43).

### Serum biochemical analysis

#### Collection of serum samples

Fish from each control and treatment group (5 fish/group) were randomly sampled after 48 and 96 hours post-experiment for biochemical analysis (44). Fish were anesthetized with 50 µL/L, and blood was collected from the caudal vein using a sterile 2 mL hypodermic syringe. The collected blood samples were transfered into sterile Eppendorf tubes without anticoagulant at 4°C overnight. Afterward, the samples were gently centrifuged at 4000 × g for 10 minutes at 4°C. The serum was carefully separated and stored at -20°C until further analysis. All procedures were performed in sterile conditions to ensure safety and accuracy.

#### Analysis of antioxidant enzymes

For tissue analyses, samples of gill, muscle, kidney, liver, and gut were excised and homogenized using a TissueLyser (Qiagen, Hilden, Germany). Homogenates were centrifuged at 10,000 rpm for 10 min at 4°C, and the resulting supernatants were collected and stored at −80°C for subsequent analyses following well-established methods with a few minor adjustments. SOD (Superoxide dismutase) activity was determined using a standardized protocol in a solution with sodium carbonate buffer (pH 10.2), enzyme extract, EDTA, and epinephrine (45). The absorbance change was carefully monitored at an OD of 480 nm using a Microplate reader (BioTek Epoch™ 2 Take-3 plate reader, USA).

The standard method outlined by Calibrone (46) was used to measure the activity of catalase (CAT) (46). This involved monitoring the breakdown of H_2_O_2_ by observing the absorbance at 240 nm. The reaction mixture included 50 mM H_2_O_2_ and 50 mM phosphate buffer at pH 7.2. To assess the reaction efficiency, absorbance was measured at 240 nm using a Microplate Reader (BioTek Epoch™ 2 Take-3 plate reader, USA), which was calibrated to 320 nm H_2_O_2_ with an extinction coefficient of 40 M^−1^ cm^−1^. The activity of catalase (CAT) is expressed as the amount of H_2_O_2_ decomposed per minute per milligram of protein, reflecting its efficiency in catalyzing the breakdown of hydrogen peroxide.

#### Serum biochemical profile and immune-stress response

Total protein levels in the serum of both the treatment and control groups were examined through an automated biochemical analyzer (Auto Analyzer, Transasia-Erba EM–200, USA). We also measured the activity levels of *Hsp70* (heat shock protein 70) and *C3* (complement c3) in the fish serum using standard protocols and reagents provided by the company. These measurements were performed using an ELISA kit (BT BioAssay, Shanghai, China) according to the manufacturer’s instructions. The final optical density (OD) was read at 450 nm with a Microplate reader (BioTek Epoch™ 2 Take-3 plate reader, USA) (47, 48). We ensured that each assay was performed in triplicate, with two independent experiments to ensure accuracy and reliability.

For cortisol analysis, a commercial ELISA kit from Bioassay Technology Laboratory in China was used according to the manufacturer’s instructions. The process began by adding 20 μL of each cortisol standard solution (concentrations of 0, 20, 50, 100, 200, 400, and 800 ng/mL) and fish serum samples to the microplate in triplicate. Separate wells were used for the recovery and linearity tests. The standard solutions and fish serum samples for the recovery test were run in duplicate. Next, 200 μl of horseradish peroxidase enzyme conjugate was added to each well. The plate was gently mixed for 10 minutes and then incubated at room temperature for 1 hour. After incubation, the solution from each well was removed by washing the plate with 400 μL of PBS three times, shaking the plate to ensure thorough washing and removing any residual drops that could interfere with assay accuracy. Then, 100 μL of TMB substrate was added to each well and incubated at room temperature for 15 minutes, allowing the enzymatic reaction to produce a color change. This reaction was stopped by adding 100 μL of 0.5 M phosphoric acid (H_2_PO_3_). The color intensity, which is inversely related to the cortisol concentration, was measured at 450 nm using a microtiter plate reader within 10 minutes of stopping the reaction.

### Total RNA extraction and reverse transcription

The total RNA was isolated using TRIzol^®^ reagent according to the manufacturer’s standard protocol. Briefly, liver tissues were collected from nine asymptomatic and symptomatic fish samples, pooled with three fish/replicate in three replicates, and immediately frozen in liquid nitrogen and stored at -80°C (49, 50). The tissue samples (100 mg) were aseptically homogenized for 15–30 s with 1 mL chilled Trizol^®^ (Sigma-Aldrich, USA) at room temperature and incubated at 20°C for 5 min. After this step, 300 μL of chloroform was added to the homogenate and mixed vigorously and incubated for 10 min at 20°C, then centrifuged for 20 min at 12,000 rpm and 4°C. The aqueous upper layer was collected in a new tube and mixed with 700 μL of isopropanol. The solution was then kept for 2 h at -20°C and centrifuged for 20 min at 12,000 rpm and 4°C. The obtained pellet was washed twice with 80% ethanol, centrifuged for 10 min at 10,000 rpm, and briefly air-dried to remove any residual ethanol. Following this, 50 μL of DEPC-treated sterile water was used to dissolve the RNA pellets, and the suspension was stored at -20°C until further analysis. To remove contamination of genomic DNA, the RNA samples were treated with RNase-free DNAse I (Thermo Scientific, India). To check the quality and concentration (ng/µL) of isolated RNA, absorbance was measured in a NanoDrop spectrophotometer (Thermo Scientific, India) at 260/280 nm. Afterward, RNA integrity was analyzed in 2% agarose gel.

The reverse transcription method was employed to synthesize cDNA using the RevertAid™ H Minus First Strand Synthesis Kit (Catalog number: K1631, Thermo Scientific, India). Briefly, 1 µL of random hexamer primer solution was combined with 1 µg of total RNA. Then, 8 µL of reaction mix was added, containing 20 units of ribonuclease inhibitor, 2 µL of 0.01 mol/L dNTP mix, 200 units of RevertAid™ H minus M-MuLV reverse transcriptase, and 4 µL of 5x reaction buffer (composed of 0.25 mol/L Tris-HCl pH 8.3, 0.25 mol/L MgCl_2_, and 0.05 mol/L DTT). The reaction was stopped after 5 minutes of heating at 70°C, and then cooled to 4°C. The cDNA samples were stored at -20°C before PCR analysis.

### Quantitative real-time PCR analysis

The expression of selected immune and stress-related genes was quantified by qPCR using gene-specific primers and *β -actin* as the reference gene (**Supplementary Table S2**). A total reaction volume of 20 µL, including 1 µL cDNA (50 ng), 10µL 2X Maxima SYBR Green/ROX qPCR Master Mix (Thermo Fisher Scientific, India), 0.5 µL of each specific primer, and 8 µL nuclease-free water, was maintained for the amplification of the target genes (51). For each biological replicate of the sample, the master mix was prepared in triplicate, with RT-qPCR for immune-related and housekeeping genes performed with a four-step amplification protocol: 10 min at 95°C (initial denaturation); 40 cycles of 15 s at 95°C, 30 s at 60°C, and 30 s at 72°C (amplification and quantification); 55-95°C (melting curve) with a 0.10°C/s heating rate and a continuous fluorescence measurement and 4°C cooling. For each primer set, a reaction mixture of a negative control was included by omitting the cDNA template. Relative gene expression was calculated using the comparative 2^−ΔΔCt method following Livak and Schmittgen (52). Amplification efficiencies were verified, and results were confirmed using the Pfaffl method (53). Statistical analyses were performed on ΔCt values using Student’s t-test, and differences were considered significant at P < 0.05.

### 3-D structure prediction and computational molecular docking

#### Homology modeling of protein structures and model validation

Protein sequence accession numbers were obtained from the NCBI database (http://www.ncbi.nlm.nih.gov/protein) and saved in FASTA format for structural prediction. To build the three-dimensional structure of the genes, namely *myd88* from *L. rohita*, Lipopolysaccharide (LPS), and O-antigen of LPS from *P. penneri*, we used the SWISS-MODEL workspace (https://swissmodel.expasy.org), a web-based integrated service specialized in protein structure homology modelling (54). Subsequently, the predicted models were validated using several validation methods, including PROCHECK, Verify3D, and ERRAT, available on the Structural Analysis and Verification Server (SAVES) (https://saves.mbi.ucla.edu/). The protein model was also assessed using the ProSA server (55). Verify3D is a tool that assesses the congruence between a three-dimensional anatomical model and its corresponding one-dimensional amino acid sequence. The validation process aims to ascertain the accuracy and reliability of the predicted models. In contrast, the PROCHECK server analyzes the stereochemical quality of the protein and assesses the overall quality of the structure compared to well-refined structures of similar resolution, as indicated by the Ramachandran (RC) plot. The ERRAT values provide the quality of a given model.

#### Secondary structure analysis and binding cavity prediction

The analysis of secondary structure of the three predicted structures involved the identification of several structural elements, including α-helices, β-turns, extended strands, β-sheets, and coils. The PDBsum tool was applied to acquire the topology diagrams for this investigation. CASTp is a web server that was utilized in this study to identify, outline, and measure geometric and topological features of predicted structures (56). The identification of the active sites within the predicted structures was conducted, accessible at http://sts.bioe.uic.edu/castp/index.html?201l. The CASTp 3.0 tool identifies several pockets containing amino acid residues that are potential facilitators of protein-protein interactions.

#### Docking computation studies

The docking simulation between the *myd88* and LPS/O-antigen was conducted using the High Ambiguity Driven protein-protein DOCKing (HADDOCK) (https://www.bonvinlab.org/software/haddock2.4/) as well as the HDOCK server (http://hdock.phys.hust.edu.cn/). The input involved the modeled files of the candidate proteins, together with predetermined default parameters. The results from the HADDOCK analysis demonstrated a higher degree of structural stability and were therefore taken for further analysis. The proposed methodology involves a three-step process for achieving energy minimization of a rigid body, followed by semi-flexible refinement in torsional angle space, and concluding with a finishing refinement in explicit solvent. Following the completion of each stage, the docked conformations are scored and ranked by the scoring function, enabling the identification of optimal conformations for further use in the next stage. The optimal docked conformers can be identified by examining the HADDOCK score, which accounts for the combined effects of van der Waals, electrostatic, desolvation, and restraint-violation energies, as well as the buried surface area. The computational docking results were visualized using the educational version of PyMOL (https://pymol.org/edu/).

#### Binding affinity and interaction analysis

The protein-protein interaction analysis of the docked structures was conducted using Ligplot+ software (57) and the PDBSum service (58). The most highly refined cluster obtained from the docked complex, as obtained from HADDOCK 2.4, was analyzed using PRODIGY (59), a protein-binding energy prediction server, in order to determine the binding affinity of the cluster. The data submitted to the servers comprises a file that adheres to the PDB (Protein Data Bank) format. The determination of interacting protein residues is achieved through the examination of hydrogen bonds, van der Waals interactions, and covalent bonds.

### Statistical analysis

The survival data were arcsine-transformed to meet the assumptions of homoscedasticity and normality. Using a statistical tool for the social sciences (SPSS) version 24.0, they were put through a one-way analysis of variance (ANOVA) and Duncan’s multiple range test. Gene expression results were represented as fold changes relative to the internal control gene (β-actin). The expression level in the control was regarded as 1.0, and thereby, the expression ratio of the treatments was expressed in relation to the control. Analysis for significant differences in expression levels between the control and treatment groups was performed using single-tailed Student’s t-tests on log-transformed data. The significance level was set at *P* ≤ 0.05.

## Results

This study aimed to comprehensively characterize *P. penneri*, an emerging multidrug-resistant (MDR) pathogen in *L. rohita*, by integrating pathogen identification, virulence assessment, antimicrobial resistance profiling, genomic analysis, microbiome evaluation, and characterization of the host immune response. The isolate was conclusively identified through biochemical assays, 16S rRNA sequencing, and phylogenetic analysis, while *in vivo* challenge experiments, hemolytic activity, histopathological damage, and dose-dependent mortality supported its pathogenic potentia. Phenotypic resistance to several clinically relevant antibiotics, together with genomic prediction of antimicrobial resistance–associated genes and virulence-related determinants, provides an integrated profile of adaptive features that may contribute to persistence and infection dynamics in aquaculture environments. Changes in cultivable gut microbial composition, alongside elevated MAR indices and shifts in virulence-associated taxa, suggest infection-associated alterations in the host microbial ecosystem. Concurrently, increased stress biomarkers and transcriptional modulation of immune-related genes indicate activation of host inflammatory and physiological stress pathways. In silico Myd88–lipopolysaccharide docking analysis offers a structural perspective on potential host–pathogen interactions; however, further functional studies are required to validate the mechanistic relevance of these findings.

### Pathogenic bacteria isolated from *Labeo rohita*

**Figure 2:**
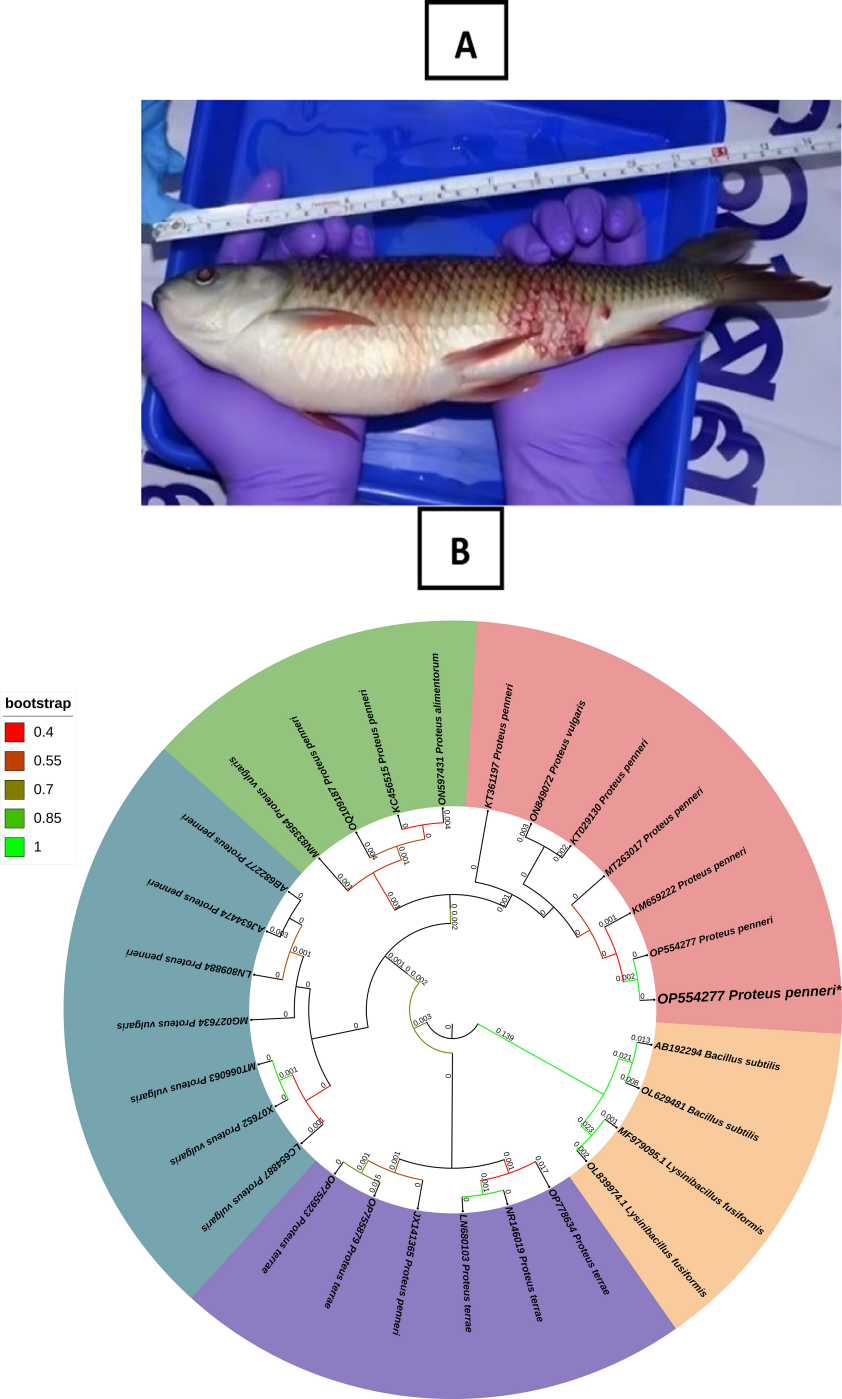
**(A)** Infected moribund *Labeo rohita* samples collected from the culture facility. **(B)** Phylogenetic tree of *Proteus penneri* based on 16S rRNA nucleotide sequences following the maximum composite likelihood method by the MEGA11 software. The numbers next to the branches indicate percentage values for 1000 bootstrap replicates. Bootstrap values above 50% are shown at the nodes. The isolates were categorized into 5 clusters indicated by color shading.

Initial signs such as ulcers, hemorrhage, discoloration, and redness across the fish’s body suggest the presence of microbial pathogens (**Figure 2A**, **Supplementary Figure S1**). The bacterial strain identified from culture was further analyzed using 16S rRNA gene sequencing and phylogenetic methods. The 16S rRNA gene sequenced was submitted to GenBank with the accession number OP554277. A BLAST search against a non-redundant database showed a perfect match (100% identity) with *P. penneri* (Accession Numbers of GenBank: KM659222 and MT263017). Subsequently, a phylogenetic tree was constructed using the sequences obtained from NCBI (**Figure 2B**). The bacteria isolated from the fish displayed characteristics of Gram-negative bacteria based on Gram staining. Biochemical testing revealed that the strain was predominantly positive for H₂S production, citrate utilization, catalase activity, glucose fermentation, indole production, urease activity, phenylalanine deamination, malonate utilization, esculin hydrolysis, arabinose, rhamnose, cellobiose, saccharose, trehalose, and glucose. The isolate showed negative results for oxidase, lactose, raffinose, Voges–Proskauer, melibiose, and adonitol (**Table 1**). Overall, the combination of sequencing and biochemical evaluations validates that the bacterial strain was *P. penneri*.

**Table 1:** Biochemical characterizations of the bacterial isolate (the activities are expressed as positive (+) and negative (-)).

Test	*Proteus penneri* (Isolated)	*Proteus penneri* (Standard)
ONPG (β-galactosidase)	+	+
Lysine	-	-
Ornithine	+	+
Urease	+	+
Phenylalanine Deamination	+	-
Nitrate	+	+
H_2_S	+	+
Citrate	+	+
Voges Proskauer’s	-	-
Methyl red	+	+
Indole	+	+
Malonate utilization	+	+
Esculin hydrolysis	+	+
Arabinose	+	+
Xylose	+	+
Adonitol	-	-
Rhamnose	+	+
Cellobiose	+	+
Melibiose	-	-
Saccharose	+	+
Raffinose	-	-
Trehalose	+	+
Glucose	+	+
Lactose	-	-
Oxidase	-	-
Catalase	+	+

Our antibiotic susceptibility findings showed that *P. penneri* is resistant to several major antibiotics, including Doxycycline, Erythromycin, Dicloxacillin, Polymyxin B, Rifampicin, Chloramphenicol, and Imipenem (**Table 2**). The bacterial strain has an elevated MAR index of 0.23, compared to other common aquatic pathogens, such as *Citrobacter freundii* (0.19), *Enterobacter cloacae* (0.19), and *Pseudomonas flexa* (0.15) (**Table 3**) (11). The MAR index results suggest that isolated *P. penneri* is a multidrug-resistant bacterium that may be able to withstand and propagate under high antimicrobial concentrations; however, this needs further validation.

**Table 2:** Antimicrobial susceptibility profile of *Proteus penneri* isolate categorized according to CLSI guidelines.

Antibiotics	*Proteus penneri* (Isolated)	*Proteus penneri* (Standard)
Trimethoprim	S	S
Netilmicin sulphate	S	S
Ampicillin	S	S
Cefixime	S	S
Tetracycline	I	S
Doxycycline	R	S
Ceftazidime	S	S
Erythromycin	R	R
Ciprofloxacin	S	S
Ofloxacin	S	S
Dicloxacillin	R	R
Nalidixic acid	S	S
Gentamicin	S	S
Amoxycillin	S	R
Polymyxin B	R	S
Colistin	S	S
Kanamycin	S	S
Tobramycin	S	S
Streptomycin	S	S
Rifampicin	I	R
Nitrofurantoin	I	S
Piperacillin	S	S
Chloramphenicol	R	I
Cefepime	S	S
Fosfomycin	S	S
Imipenem	R	S

Following the guidelines of the Clinical and Laboratory Standards Institute (16, 17), susceptibility of recovered strains to different antibiotics is expressed as sensitive (S), intermediate (I) and resistant (R).

**Table 3:** MAR indices of the isolated bacterial strain.

Bacterial strains	MAR value
*Proteus penneri*	0.23

### Genome assembly and annotation

**Figure 3:**
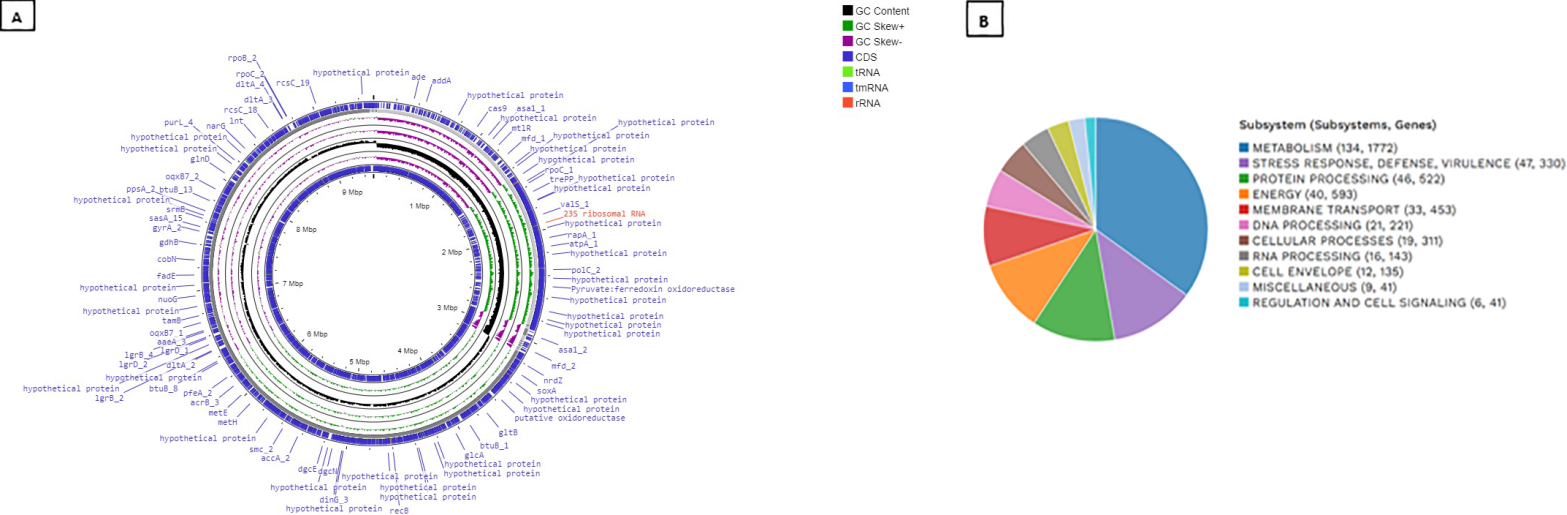
**(A)** The circular genome of *Proteus penneri* has been illustrated. The figure represents various genomic annotations, arranged from outside to inside, such as contigs, CDS on the forward and reverse strands, tRNA, CDS of AMR genes, CDS of virulence factors, GC content, and GC skew. **(B)** Distribution of subsystem category for *P. penneri* and strains organized via pattern Subsystem Counts (Subsystems, Genes).

A thorough genomic study of the *P. penneri* species was conducted. *De novo* assemblies were prepared by the Canu assembler, and assembly statistics were performed by QUAST and BUSCO (**Supplementary Figure S2**). The genomic assembly revealed 20 contigs, a genome length of 9,532,803 bp, and a G+C content of 56.91%. The N50 length, which represents the shortest sequence length of the genome at 50%, was 6372589 bp. Genome completeness assessment using BUSCO indicated 94% complete BUSCOs, with a substantial fraction categorized as duplicated (63%), alongside 25% fragmented and 5% missing BUSCOs (**Table 4**; **Supplementary Figure S2**). Because BUSCO marker genes are expected to be largely single-copy, an elevated duplicated fraction can reflect assembly redundancy, unresolved repeat structure, or mixed-strain content rather than true biological duplication. Therefore, these genome statistics were interpreted in conjunction with assembly-level quality control metrics when evaluating downstream comparative genomics and resistome annotations. An analysis of the genome annotation for the *P. penneri* species (**Figure 3A**) revealed that it contains 9240 coding DNA sequences (CDS), 135 transfer RNA genes (tRNA), and 24 ribosomal RNA genes (rRNA). The annotated features are summarized in **Table 4** below. The functionally assigned proteins encompassed 2,221 proteins with EC (Enzyme Commission) numbers (60), 1,837 with GO (Gene Ontology) classifications (61), and 1,610 proteins linked to KEGG pathways (31). The PATRIC annotation system incorporates two categories of protein families (26). Within this genome, 841 proteins are associated with genus-specific protein families (PLFams), whilst 9,637 proteins belong to cross-genus protein families (PGFams) (**Table 5**). The whole-genome sequence of *P. penneri* was further analyzed using Prokka (**Supplementary Figure S2**).

**Table 4:** Genome assembly and annotation details of *Proteus penneri*.

Bacterial isolate	*Proteus penneri*
Sequencing method	PacBio sequel II
Hifi reads	17543773
Length of contig	9531409
No. of contigs	20
Total length (bp)	9531409
N50	6372589
GC%	56.91
Contamination	–
Sequencing coverage	55.73
Complete BUSCOs (C)	94
Complete and single-copy BUSCO (S)	31
Complete and duplicated BUSCOs (D)	63
Fragmented BUSCOs (F)	25
Missing BUSCOs (M)	5
No. of CDSs	9240
No. of rRNAs	24
No. of tRNAs	135
No. of tmRNAs	2
Antimicrobial Resistance Genes (total)	205
BioProject number	PRJNA1216581
Biosample accession no.	SAMN46437206
SRA number	SRR32167595

**Table 5:** Genome annotation details of *P. penneri* with their functional details.

Protein Features	
Hypothetical proteins	2461
Proteins with functional assignments	7647
Proteins with EC number assignments	2221
Proteins with GO assignments	1837
Proteins with Pathway assignments	1610
Proteins with PATRIC genus-specific family (PLfam) assignments	841
Proteins with PATRIC cross-genus family (PGfam) assignments	9637

### Antimicrobial resistance candidate and other genes

Numerous genes identified in the annotation process exhibit sequence similarity to known transporters, virulence factors, potential drug targets, and genes linked to the antibiotic resistance profile. Details on the gene numbers and the specific databases in which homology was found are listed in **Supplementary Table S3**. AMR annotation was performed using the BV-BRC/PATRIC framework, which applies a k-mer–based homology detection approach against curated resistance gene datasets. Identified genes were classified into broad resistance-related functional categories, including efflux systems, enzymatic modifiers, and regulatory components commonly associated with intrinsic or adaptive resistance in Gram-negative bacteria. It is important to emphasize that homology-based identification of AMR-associated genes does not necessarily equate to phenotypic resistance. The functional expression of resistance depends on gene regulation, genomic context, copy number, and specific mutations that may alter activity. Therefore, the predicted resistome profile should be interpreted as a genomic assessment of resistance potential rather than direct confirmation of antimicrobial resistance. A comprehensive overview of the annotated AMR-related genes and their predicted resistance mechanisms is provided in **Supplementary Table S4**.

### Subsystems in *P. penneri*

A subsystem comprises a group of proteins that collaborate to perform a specific biological function or form a structural complex. The circular diagram illustrates the characteristics of each subsystem and its respective coverage. Various genes have been assigned to various subsystems, with amino acid metabolism receiving the largest allocation (134, 1772). This organization follows the pattern of Subsystem Counts (Subsystems, Genes), indicating that in the case of *P. penneri* metabolism, it represents a particular biological process involving 134 subsystems regulated by 1772 genes. The subsystems overview for this genome is provided in **Figure 3B**.

### Phylogenetic analysis

Representative and reference genomes curated by NCBI were retrieved from the PATRIC database and included in the phylogenetic analysis within the Comprehensive Genome Analysis framework. Genome selection was based on publicly available reference and representative status annotations. Mash/MinHash was employed to identify the most closely related reference and representative genomes (62). To determine the phylogenetic position of this genome, PATRIC global protein families (PGFams) were chosen from these genomes (26). MUSCLE was used to align the protein sequences from these families, and we carefully mapped the corresponding nucleotides to the protein alignment to ensure accuracy. The combined amino acid and nucleotide alignments were merged into a data matrix, which was then analyzed using RaxML (29). Fast bootstrapping was employed to generate the support values in the resulting tree (**Supplementary Figure S3**). TYGS analysis also yielded similar results, with the strain most closely related to *Enterococcus* sp.

### Functional annotation of *P. penneri*

To further investigate the functional landscape of the annotated genome, Gene Ontology (**Supplementary Figure S4A**) and KEGG pathway mapping analyses were performed on the predicted resistance-associated genes. Functional categorization revealed enrichment of genes annotated within pathways related to antimicrobial resistance, including β-lactam resistance, cationic antimicrobial peptide (CAMP) resistance, and other drug-associated response pathways (**Figure 3B**).

### *P. penneri* displays high virulence and induces histopathological changes

**Figure 4:**
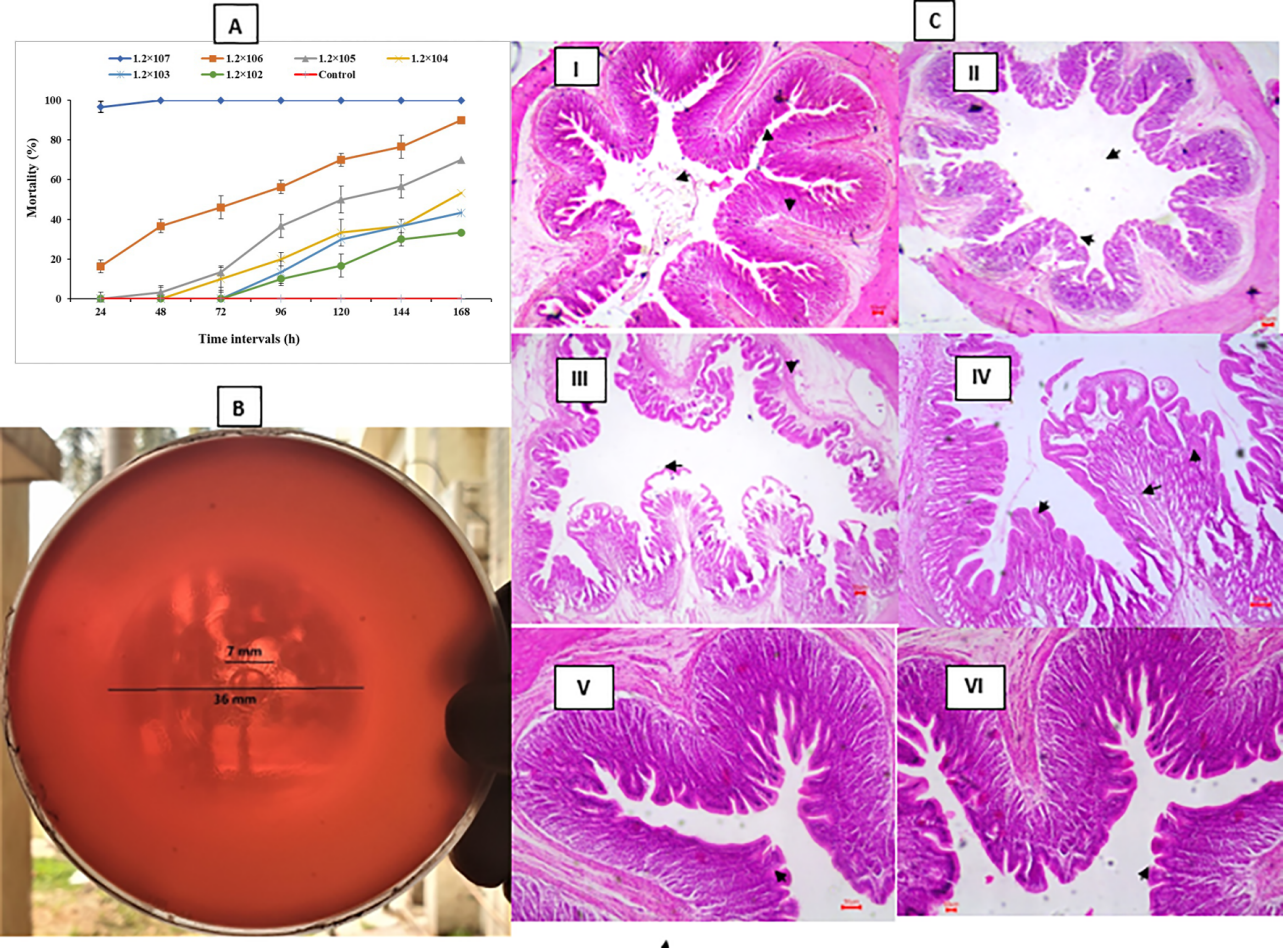
**(A)** Cumulative mortality (%) of *Labeo rohita* fingerlings recorded up to 168 h following intraperitoneal challenge with Proteus penneri. Fish were injected with 1.2 × 10^2^, 1.2 × 10^3^, 1.2 × 10^4^, 1.2 × 10^5^, 1.2 × 10^6^, or 1.2 × 10^7^ CFU mL^-1^ of bacterial suspension. Control fish received 200 µL sterile saline. Fish were maintained in FRP tanks and monitored every 24 h for 168 h. Error bars represent the standard error of five replicates. **(B)** Hemolytic activity of *P. penneri* on blood agar plates, showing a clear hemolysis zone surrounding the bacterial colony. **(C)** Histopathological alterations in the intestinal tissue of *L. rohita* following experimental infection with *P. penneri*. (I–II) At 48 h post-infection, the treatment group exhibited shortened and irregularly shaped intestinal villi. (III–IV) At 96 h post-infection, pronounced necrosis and destruction of intestinal villi were observed. (V–VI) Control fish displayed normal intestinal architecture with intact epithelium and well-organized villi at both 48 h and 96 h. Arrowheads indicate pathological changes associated with P. penneri infection. Scale bar = 100 µm; magnification = ×100.

We then explored the virulence of the isolated *P. penneri* strain, as it may play a role in disease development and mortality in *L. rohita* during infection. The overall mortality rates of *L. rohita* after exposure to *P. penneri* are shown in **Figure 4A**. Fish injected with 1.2 × 10^7^ CFU/mL showed a 100% mortality rate, while those given 1.2 × 10^6^ had more than 80% mortality. Most of the challenged fish developed subcutaneous hemorrhagic ulcers measuring about 0.6-1.7 cm across. Redness at the injection sites and ulcers around the mouth were also seen. Fortunately, the control fish did not exhibit any mortality or signs of infection during the experiment. The bacteria were re-isolated from the blood, liver, and kidney of the challenged fish and confirmed as *P. penneri*. The hemolytic proteins produced by this pathogenic strain are key virulence factors that work by forming membrane-spanning pores from identical subunits. Alongside the survival test, the results demonstrated that the *P. penneri* strain had notably higher hemolytic activity on blood agar (**Figure 4B**). Overall, these findings suggest that the *P. penneri* strain isolated from *L. rohita* could be involved in the infection process, leading to a high mortality rate among the fish.

The histological analysis reveals that *P. penneri* infection causes varying degrees of cellular changes in the gut tissue of *L. rohita*. The results showed that at 48 h post-experiment, the intestinal layer of infected fish appeared pale and filled with mucus in the lumen. The pathological effect exhibited a ruptured serosa, shortened and irregularly shaped villi (**Figure 4C**I, II). While at 96 h post-experiment, breakage, hemorrhage, and separation of villous processes with large spaces appeared in the gut tissue of challenged fish (**Figure 4C**III, IV). Necrosis and destruction of villi were also observed during 96 post-experiments. Furthermore, the control group exhibited typical gut tissue histology, with an intact epithelium and normal villi (**Figure 4C**V, VI).

### Effect of *P. penneri* on host gut microbiota

The gut microbiota is essential for processing nutrients, maintaining gut lining health, supporting immune function, and safeguarding against harmful microbes. It’s also thought to be important in controlling dangerous pathogens by limiting their growth and fighting them off with antimicrobial activity. Furthermore, pathogens have developed strategies to enhance their replication despite the presence of gut microbiota. To gain a better understanding of *P. penneri’s* pathogenesis, we selected our sampling approach by isolating gut bacteria with diverse features, such as color, shape, and size, which help identify different bacterial groups. We also used a designed culture method to target the bacterial species present in the gut samples from *L. rohita*. We obtained a total of 24 axenic bacterial strain cultures from infected and control gut samples at 48 and 96 post-experiment (**Supplementary Table S5**).

Next, we evaluated the isolates’ phylogenetic diversity using 16S rRNA amplicon sequencing data. This approach provided initial insights into the taxonomic clades of the bacterial isolates, revealing that most were Gram-negative, facultative anaerobes belonging to the Enterobacteriaceae family. Moreover, there were bacterial strains from the Gram-positive and aerobic groups, belonging to the Morganellaceae and Staphylococcaceae families. The isolated bacteria included 9 *Proteus* species, 6 *Citrobacter* species, 5 *Providencia* species, 2 *Staphylococcus* species, and 2 *Plesiomonas* species. Most bacterial isolates clustered within the Enterobacteriaceae family, which is considered a normal component of fish microbiota. However, few genera within this family have been linked to major disease outbreaks. Notably, we also detected several members of the Morganellaceae family (*Providencia aicalifaciens*) and the Staphylococcaceae family (*Staphylococcus epidermidis*) in the gut samples of *L. rohita* (**Supplementary Table S5**). The fish challenged with *P. penneri* has the highest abundance of *Proteus* species, followed by *Citrobacter* and *Providencia* species. Meanwhile, *Providencia* is the dominant genus in the control group, followed by *Citrobacter*, *Plesiomonas*, *Staphylococcus*, and *Proteus*. Furthermore, this study examined only a small subset of the extensive bacterial community, and there are likely additional microorganisms influenced by *P. penneri* in *L rohita*.

### Antibiogram assay and virulence characteristics of gut bacterial isolates

The antibiotic resistance profile of bacterial isolates from infected *L. rohita* gut samples revealed that the isolates were resistant to multiple tested antibiotics (**Supplementary Table S5**). Approximately 50% of the isolated bacteria from infected fish gut samples at 48 and 96 h post-experiment had a MAR index ≥0.15, with values ranging from 0.11 to 0.24. Moreover, the isolates from control fish gut samples have the lowest MAR index values, ranging from 0.11 to 0.19 (**Supplementary Table S7**).

Next, we aimed to explore the virulence mechanism of the bacterial isolates through analysis of hemolysin activity and survival assay. One key bacterial virulence factor is the production of hemolytic proteins, often found in pathogenic bacteria. These proteins work by combining identical subunits to form a membrane-spanning pore. Results from the hemolysin test showed that bacterial isolates from treated *L. rohita* samples had significantly increased hemolytic activity. In contrast, isolates from control *L. rohita* samples had little to very negligible or no effect on blood agar, except for three bacterial isolates that showed higher hemolysin activity (**Supplementary Tables S6**, **S7**). Meanwhile, the isolated bacterial strains from infected *L. rohita* gut samples had the most pathogenic bacterial isolates. A survival study found that over 90% of bacterial isolates from infected fish samples containing *P. alcalifaciens*, *C. amalonticus*, *P. penneri*, and *P. terrae* caused significantly high mortality in *L. rohita* fingerlings. On the other hand, bacterial strains isolated from control fish gut samples were non-pathogenic primarily, with only three bacterial isolates causing significantly high mortality (*P. alcalifaciens*, *P. penneri*, and *Plesiomonas shigelloides*) in *L. rohita* over 24–168 hours (**Supplementary Table S8**).

### Effect of *P. penneri* on antioxidant defense, health and immunity of *L. rohita*

**Figure 5:**
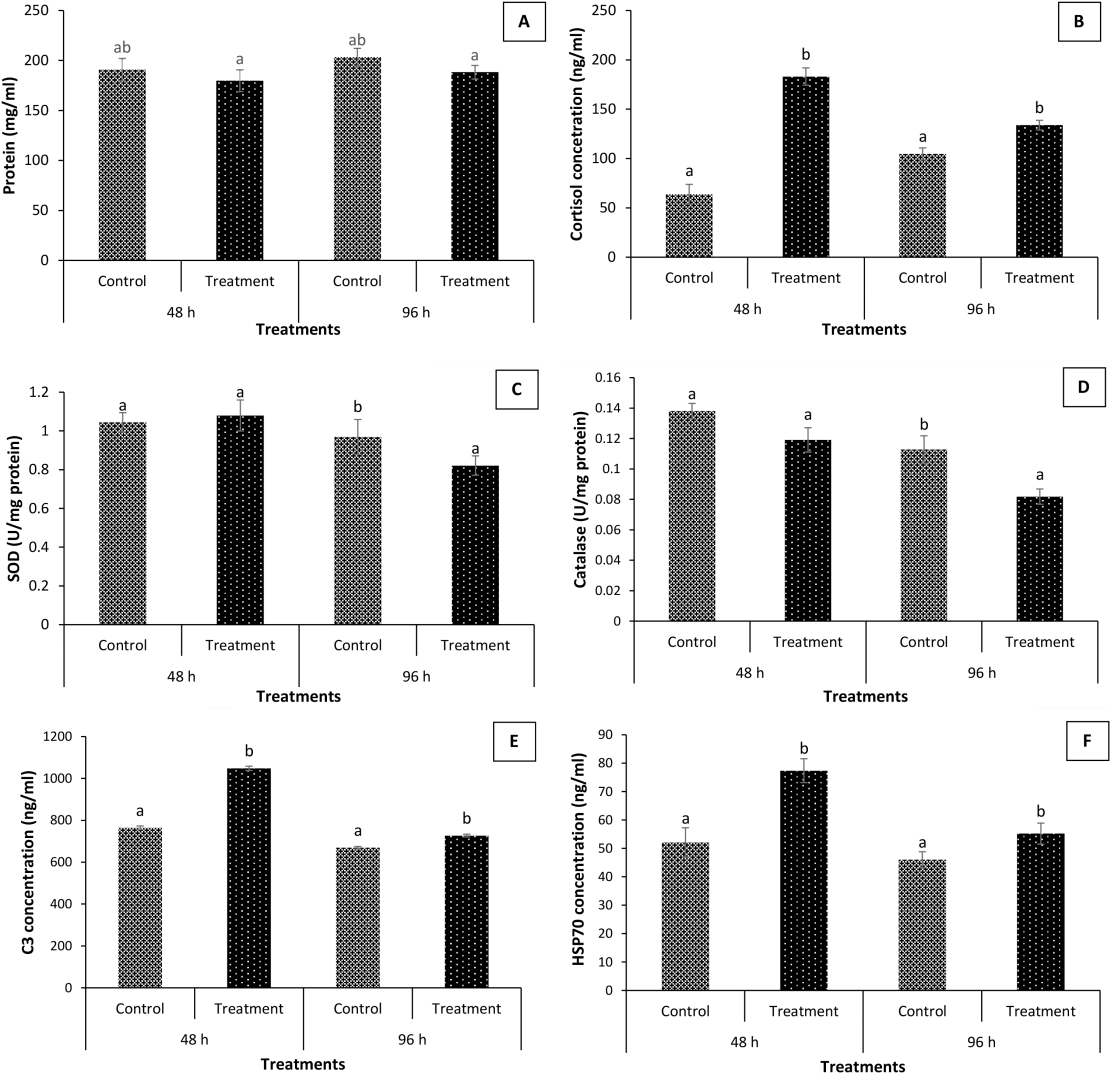
Effect of *Proteus penneri* infection on total protein level, stress and antioxidative response and immune response of *Labeo rohita*. The serum samples of *L. rohita* fingerlings were collected from control and treatment groups after 48 and 96 h post-challenge. **(A)** total protein (mg/L); **(B)** cortisol concentration (ng/mL); **(C)** superoxide dismutase (SOD) activity (U/mg protein); **(D)** catalase (CAT) activity (U/mg protein); **(E)** C3 concentration (ng/mL) and **(F)** HSP70 concentration (ng/mL). Error bars represent the standard error of five replicates; different letters indicate significant differences (*P* < 0.01).

In the next experiment, we explore how *P. penneri* causes disease by examining the antioxidant defenses, health, and immunity of *L. rohita*. We found that total protein levels were similar in both the treatment and control groups, and remained at baseline in the fingerlings. The protein level ranged from 190.86 ± 11.2 to 203.3 ± 8.8 ng/mL in the control, while in the treatment, values of 179.75 ± 10.9 and 188.2 ± 6.8 ng/mL were recorded at 48 and 96 h post-experiment (**Figure 5A**). The control group’s fingerlings exhibited cortisol levels at a basal or resting state. Additionally, there were significant positive correlations between *P. penneri* infection and cortisol levels in *L. rohita*. The fingerlings in the treatment group exhibit notably higher serum cortisol levels. Interestingly, *P. penneri* infection significantly increased cortisol levels, with high values observed at both 48 and 96 h post-experiment (**Figure 5B**). In contrast, the activity of SOD and CAT was significantly decreased following *P. penneri* infection at 48 and 96 h post-experiment. The lowest values were recorded in treatment groups at 96 h post-experiment (**Figures 5C, D**). Later, we used enzymatic assays to assess the role of *P. penneri* infection on non-specific immunity (C3 and HSP70) of *L. rohita*. We found that *P. penneri* infections resulted in significantly increased levels of C3 and HSP70 in fish serum compared to the control group (**Figures 5E, F**). The highest levels of C3 and *HSP70* activity were observed in the treatment groups at 48 h, followed by 96 h post-experiment.

**Figure 6:**
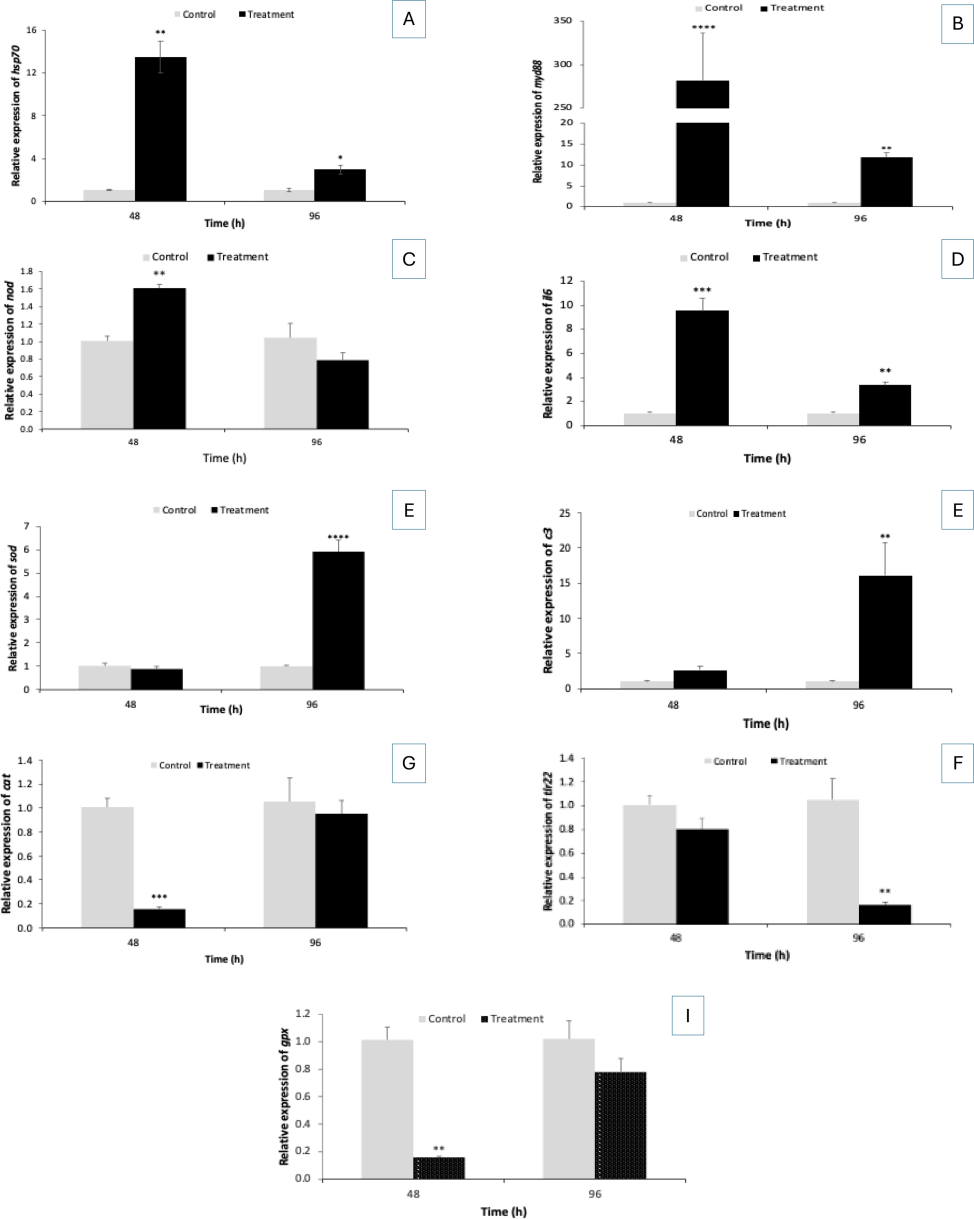
The expression of **(A)** heat shock protein 70 (Hsp70); **(B)** Myeloid differentiation primary response 88 (MyD88); **(C)** Nucleotide-binding oligomerization domain (NOD); **(D)** Interleukin-6 (IL-6); **(E)** superoxide dismutase (SOD); **(F)** complement factor 3 (C3); **(G)** catalase (CAT); **(H)** Toll-like receptor (*tlr22*) and **(I)** Glutathione peroxidase (GPX) genes measured at 48 and 96 h post experiment from control and treatment *L. rohita*. The expression in the control samples of *L. rohita* group at 48 and 96 h post-experiment was set at 1. The results are the mean ± standard error (n = 3). Asterisks represent a significant difference between the control and treatment *L. rohita* group. *P < 0.05, **P < 0.01, ***P < 0.001, ****P < 0.0001, *****P < 0.00001.

Since bacterial pathogenesis is closely linked to fish health, the study explored how the expression of various important genes, like *hsp70* (heat shock protein 70), *sod* (superoxide dismutase), *cat* (catalase), *c3* (complement factor 3), *tlr22* (toll-like receptor22), *nod* (nucleotide-binding oligomerization domain), *gpx* (glutathione peroxidase), *il-6* (interleukin-6), and *myd88* (Myeloid differentiation primary response 88), changes over time at the transcriptional level *in vivo*. The results showed that the expression of *hsp70* (~13 folds), *myd88* (~280 folds), *nod* (~1.6 folds), and *IL-6* (~9 folds) genes was significantly upregulated in the infected *L. rohita* group compared with the control fish at 48 h, while downregulation or lower transcription values were recorded at 96 h post-experiment (**Figures 6A–D**). Moreover, the *sod* (~6-fold) and *c3* (~15-fold) gene expressions were significantly increased in the infected L. rohita group at 96 h, with the lowest values recorded at 48 h post-experiment (**Figures 6E, F**). In contrast, the transcription of *cat*, *tlr22*, and *gpx* was significantly downregulated in the infected group compared with the non-infected control *L. rohita* group. The lower values were recorded in both 48 and 96 h post-experiment (**Figures 6G-I**). Although immune gene expression varies, the results show a meaningful interaction between *P. penneri* infection and immune components. It appears that bacteria may be suppressing specific immune cells to establish a foothold in the host, and vice versa. This intriguing possibility requires further validation.

### Interaction of *myd88* protein with bacterial lipopolysaccharides and O-antigen

**Figure 7:**
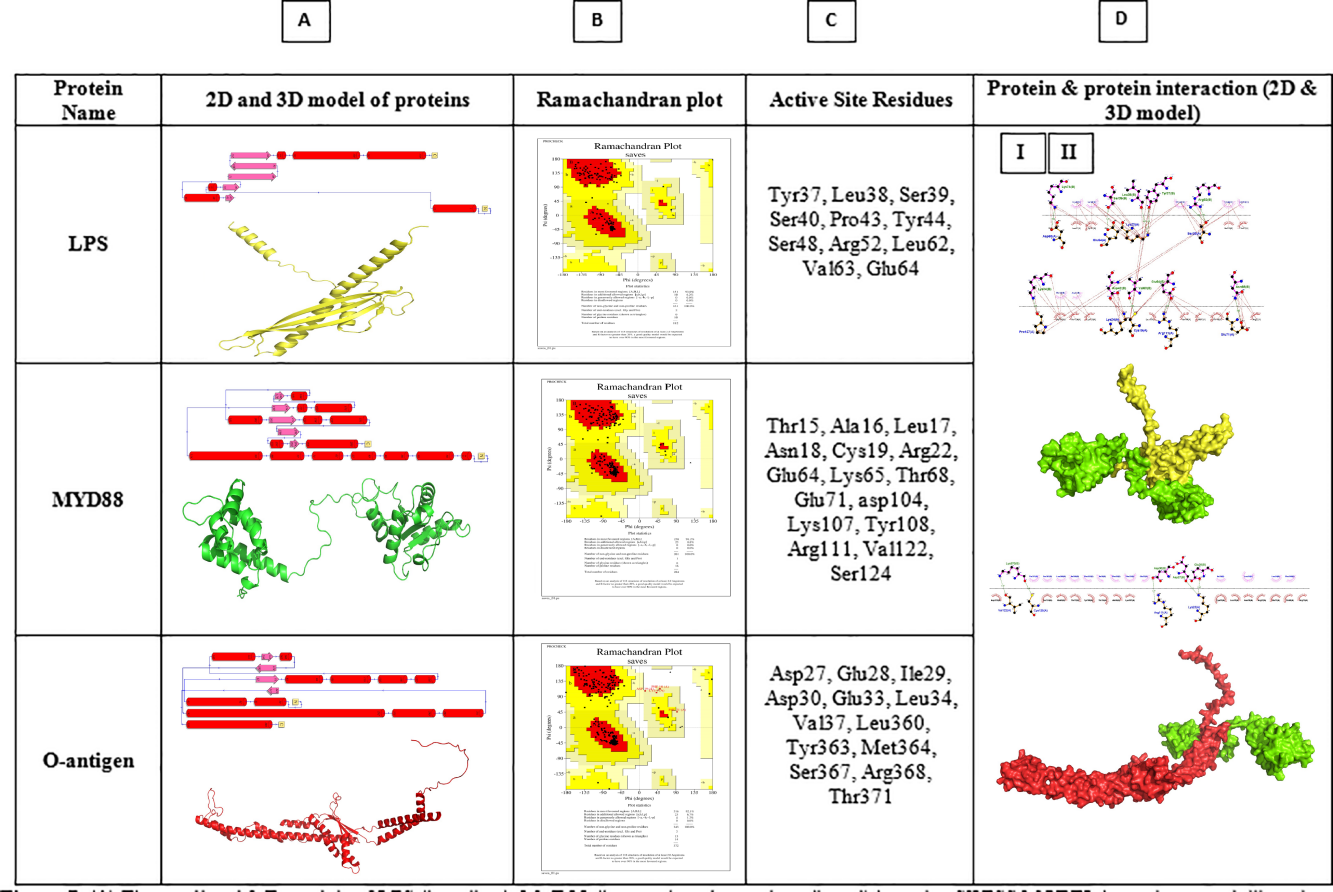
**(A)** The predicted 3-D models of LPS (in yellow), *Myd88* (in green) and o-antigen (in red) by using SWISS MODEL homology modelling along with the schematic diagrams showing the secondary structural elements calculated using PDBSum tool; **(B)** Result of the 3-D structure validation using Ramachandran Plot analysis as obtained from PROCHECK; **(C)** The identified active site residues of the three structures calculated using the CASTp tool; **(D)** The best structure of *Myd88*-LPS (I) and *Myd88*-o-antigen (II) docked complex, as depicted through molecular surface representation. *Myd88* is represented in green in both the figures, and LPS and O-antigen are represented in blue in the two figures. The interaction analysis of the docked structures, showing the amino acid residues interacting via polar (hydrogen) and hydrophobic contacts, is depicted through the Ligplot+ illustrations for the *Myd88*-LPS (I) complex and MyD88-o-antigen (II) complex.

A correlation exists between bacterial pathogenesis and the protective immune response in host animals. Hence, to gain insight into the molecular bases of the previously observed effects, we used molecular docking studies to assess the role of the significantly upregulated *myd88* protein in protecting *L. rohita* against Lipopolysaccharides (LPS) and O-antigen, which are responsible for bacterial virulence. One advantage of protein/gene structure modeling is its pivotal role in investigating the association between these proteins (**Supplementary information**). The predicted 3-D protein structures were generated using the SWISS-MODEL server (**Figure 7A**). Upon examination of the Ramachandran plot, the model that exhibited the highest level of optimality was selected from a range of structures for MyD88, LPS and O-antigen. The ideal model was chosen based on the highest percentages of residues detected in the most preferred regions and the lowest percentage scores in the outlier region. The results revealed that for *Myd88*, 91.2% of the residues were in the most favored region, 8.8% in the additionally allowed region, with no residues in the generously allowed and disallowed regions (**Figure 7B**). In the case of LPS, the Ramachandran plot revealed that 93.8% of the residues were in the most favored region, 6.2% in the additionally allowed region, with no residues in the generously allowed and disallowed regions (**Figure 7B**). Similarly, for the O-antigen, the Ramachandran plot revealed that 92.1% of the residues were in the most favored region, 6.7% in the additionally allowed region, and 1.2% in the generously allowed region, with no residues in the disallowed regions (**Figure 7B**). Subsequently, an evaluation was performed to determine the quality of the protein structure predicted using the SAVES server. The scores obtained from ProSA, Verify3D, and ERRAT for the projected models of each structure are listed in **Supplementary Table S9**.

The PDBsum tool was then used to perform a thorough examination of the secondary structural components. The secondary structures or topology maps, as depicted in **Figure 7A**, were generated using the PDBsum tool, which provides information on the number of alpha helices, beta sheets, and coils observed. The secondary structure of MyD88 is characterized by the presence of 15 helices involved in a total of 16 helix-helix interactions and exhibits 5 beta-sheet motifs. On the other hand, LPS and the O-antigen possess 6 α-helices engaged in 3 helix-helix interactions and 5 β-sheets. O-antigen possesses 12 helices with 14 helix-helix interactions and 4 beta-sheets. The CASTp tool was used to identify the amino acid residues present in the active sites of the three modelled protein structures. The analysis revealed a single pocket at the active sites of all three structures. The pockets selected for the molecular docking studies on the protein are shown in **Figure 7C**.

HADDOCK 2.4 was used to perform molecular docking between the protein structures (**Supplementary information**). In the case of the docking between *Myd88*-LPS (**Figure 7D**I), the most reliable cluster obtained from HADDOCK had the lowest score of -85.6 +/- 2.0 a.u. and Z-score −1.6. For *Myd88*-O-antigen (**Figure 7D**II), the most reliable cluster had HADDOCK score -104.6 +/- 7.2 a.u. and Z-score (−1.7). The docking analysis includes several parameters: HADDOCK score, RMSD, binding energy, van der Waals energy, electrostatic energy, desolvation energy, and restraint violation energy. Negative scores indicate a favorable binding affinity between proteins, suggesting robust, reliable structural interactions. The 3D models of the docked complexes are represented in figures (**Figures 7D**I-II), where the green color represents *Myd88* in both, while the blue represents LPS in **Figure 7D**I and O-antigen in **Figure 7D**II.

**Figure 8:**
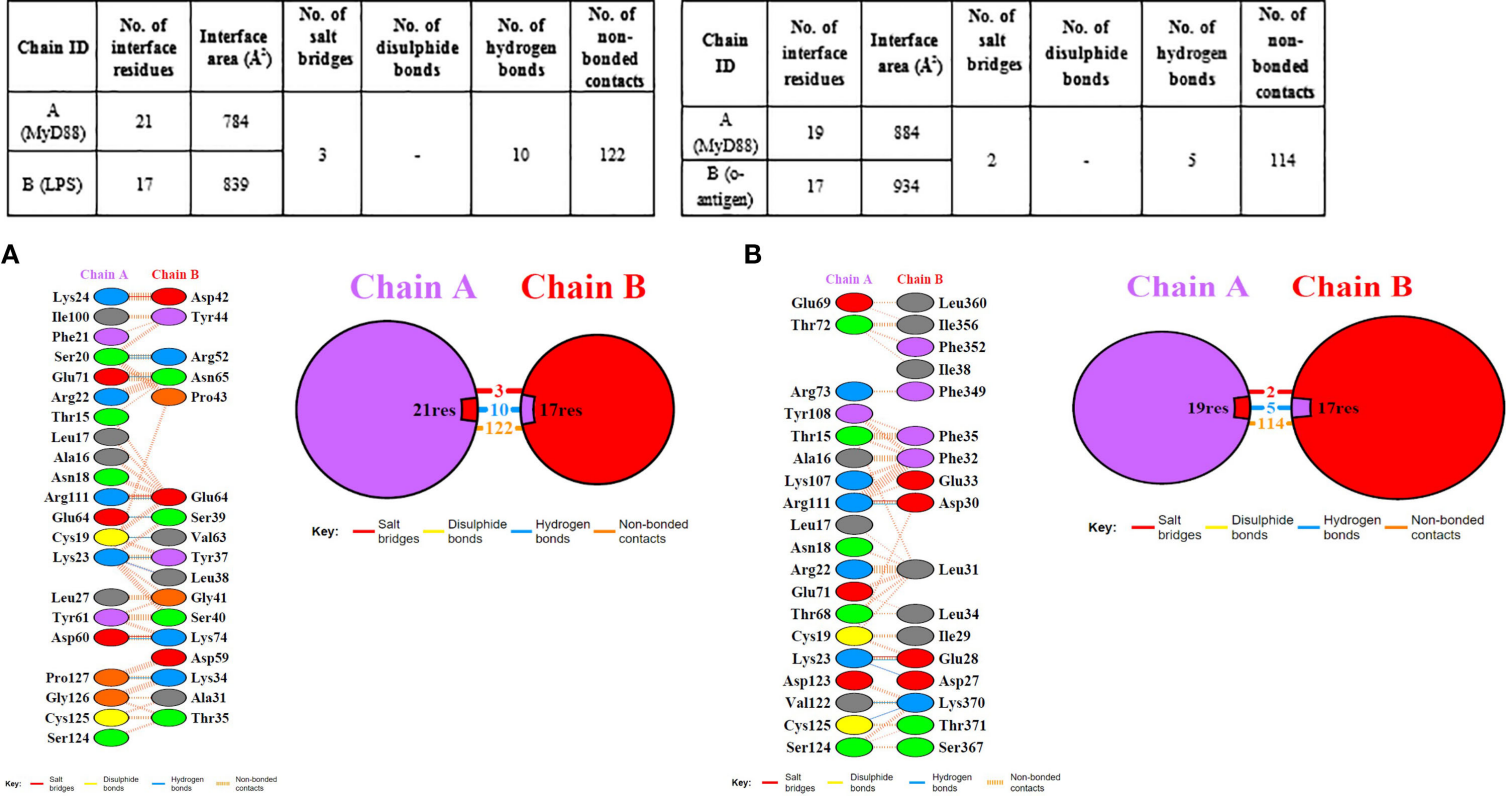
Protein-protein interaction analysis of the docked complexes as obtained from PDBSum showing the interface statistics with the number of polar contacts, surface area, non-polar contacts and the interacting residues of **(A)***Myd88*-LPS and **(B)***Myd88*-o-antigen docked complexes.

The protein-protein interaction analysis was carried out using the PDBsum server (58) (**Figures 8A, B**) as well as Ligplot+ (57) (**Figures 6D**I, **7D**II), which gave us the interacting residues in the docked structures. Moreover, the determination of binding affinities of complexes, as indicated by the Gibbs free energy (ΔG) in thermodynamics, plays a crucial role in assessing the likelihood of an interaction under specific biological conditions. The binding constant offers valuable information regarding the equilibrium binding affinity between two compounds. The negative G values observed serve as strong evidence for each of the docked complexes. Here, we applied the PRODIGY (59) server and found the ΔG to be -9.8 kcal/mol and K_d_ to be 6.1e-08 M for the interaction between *Myd88*-LPS, whereas for *Myd88*-o-antigen, ΔG is -10.8 kcal/mol and K_d,_ 1.3e-08 M.

## Discussion

The increasing occurrence and severity of fish disease outbreaks pose significant risks to aquaculture, global food security, and biodiversity loss in many fish-producing countries. These disease outbreaks cause production losses, directly affecting regional economies and other interconnected socio-economic aspects (63). Furthermore, human-caused climate change increases the risk of fish diseases, threatening the world’s food supply and natural aquatic biodiversity. It is believed that any potential increase in yield over the next fifty years will be offset by climate change-induced shifts in disease pressure from both existing and emerging pathogens (64). Likewise, the spread of pathogens linked to climate change is considered a significant threat to fish health worldwide (65). Therefore, gaining better knowledge of how pathogens interact with fish is essential for developing a climate-resilient aquaculture system. This motivated us to develop an infection model using a highly pathogenic bacterial strain to delineate the changes in host health and immunity. We isolated a bacterium, *P. penneri*, from moribund *L. rohita*, which is suspected of being associated with mortality in the fish. We found that the *P. penneri* strain indeed could cause high mortality and histological changes in *L. rohita*. The bacterium exhibits high hemolysin activity and multiple antibiotic resistance (MAR) profiles. Subsequent studies were conducted to elucidate the host-pathogen relationship, and the results showed that the *P. penneri* strain modulates the gut microbiota, with more pathogenic and MAR bacterium colonizing the infected fish species. Additionally, *P. penneri*-induced stress conditions decreased the antioxidant response, while host-generated immunity may play a crucial role in neutralizing bacterial virulence and in developing a protective immune response in the host. However, it would be especially useful to identify the immune cells involved in host resistance, which would facilitate the targeted development of future management measures.

Although many biological, ecological, and environmental factors play a role in the emergence of a disease-causing pathogen, at its most fundamental level, it relies on the interaction between a pathogen and a fish. The outcome depends on how well the fish’s resistance to stressful conditions holds up, or whether a climate-related variable increases the pathogen’s strength. Moreover, the increased morbidity and mortality rates associated with infections are often related to antibiotic-resistant bacteria. The aquaculture sector plays a significant role in the AMR reservoir mainly through the use of therapeutic and preventive antimicrobial treatments given to animals. Additionally, the use of non-antibiotic chemicals, such as disinfectants, which have been shown to contribute to AMR, further exacerbates this issue (66). The presence of AMR in aquaculture not only poses a direct risk to human health but can also affect production by reducing the effectiveness of drugs, weakening animals’ immune systems, and encouraging the emergence of more aggressive strains that grow faster and spread more easily. In our study, we isolated a pathogenic bacterium, *P. penneri*, exhibiting high virulence, as evidenced by hemolysin activity, survival assays, and histological alterations in the host fish. Additionally, the bacterium was resistant to different major antibiotics, including Doxycycline, Erythromycin, Dicloxacillin, Polymyxin B, Rifampicin, Chloramphenicol, and Imipenem, with a MAR index of 0.23. The findings suggest that the emergence of pathogenic and antimicrobial resistance (AMR) in *P. penneri* in aquaculture systems may pose a significant threat to fish health, highlighting the need for surveillance to quantify the spread and associated risks of the bacterium (67).

Identifying genetic characteristics through genomic annotation and comparative genome analysis of *P. penneri* is crucial. Our study found that the annotation of the *P. penneri* strain exhibited remarkable resemblances in numerous aspects to those previously documented in studies (68, 69). Examining the whole genome thus enables us to obtain a thorough grasp of the genetic composition and evolutionary patterns of the strain species. The thorough comparison encompassing genome feature annotation, antimicrobial resistance gene (AMR) identification, and phylogenetic relationship analysis, provides insight into the genetic makeup of this pathogen. The presence of AMR in the *P. penneri* species suggests that they employ various mechanisms to combat antimicrobial agents. One such mechanism is the intracellular nature of *P. penneri*, which impedes the penetration of certain antimicrobials into cells, thus fostering resistance development. This investigation thus enhances our knowledge of *P. penneri* evolution in farmed *L. rohita* by providing insights into genetic differences, possible factors influencing its severity, and links to vaccines for this pathogen.

How pathogenic microbes interact with the host’s gut microbiota is a complex and ongoing area of research, reflecting both the immune response and overall fish health (70). When microbes infect, they can significantly alter the gut environment by releasing toxic metabolites that change conditions, affect the growth of other organisms, and disrupt the balance of microbes (71). The “selfishness” of the winning species also impedes coexistence, thereby reducing biodiversity. Active growth can lead to competition for vital nutrients, which helps counterbalance the harsh effects of strong competitors on the ecosystem (72). By studying the relationship between microbial infection and the host’s gut microbiota, we may identify biomarkers that influence fish growth and survival. In our study, we found that the presence of *P. penneri* had a significant impact on the microbial community structures. The infected fish has the highest abundance of *Proteus* species, followed by *Citrobacter* and *Providencia* species. In contrast, *Providencia* species is the dominant genus in the control group, followed by *Citrobacter*, *Plesiomonas*, *Staphylococcus*, and *Proteus* species. It shows that *P. penneri* infections drive community succession forward in *L. rohita*. In parallel, Zhou et al. (73) reported that bacterial infection disrupts the intestinal bacterial community and facilitates the enrichment of pathogenic bacteria in the intestines of aquatic species.

What makes these analyses unique is their ability to capture interactions at the systems level. This approach allows us to examine not only the overall system but also how individual components influence and respond to one another. For example, in studying the impact of bacterial infection on gut microbial phenotypes, we observed that key traits, such as antibiogram profiles, biofilm formation, and virulence across different bacterial species, provide valuable insights into microbial behavior. These insights are critical for informing the development of effective treatment strategies and infection-control measures. Similarly, stress in fish can alter the gut environment by promoting colonization by distinct microbial communities. These microbial shifts may also be linked to the development of chemotherapy resistance, underscoring the complex and influential role of gut microbiota in host health (16, 17). Researchers have known about growing antibiotic resistance in bacteria for decades (74). Recently, the bacterial habitat’s microenvironment has been identified as a key hotspot for the spread of antibiotic resistance. When bacteria are exposed to environmental chemicals or pollutants, it can increase the diversity of resistance genes in different forms, such as resistant bacteria that can share genetic material, free plasmids or DNA, and phage particles. This makes it more likely for genes to be transferred within the bacterial community. (75). Our research revealed high levels of antibiotic resistance markers in the gut microbiota of *L. rohita* infected with bacteria. We found that many isolates showed resistance to multiple antibiotics. The unusually high MAR indices suggest that *L. rohita* infected with *P. penneri* may carry multidrug-resistant bacteria. This is a significant concern because bacterial infections are common in fish; the presence of MDR bacteria in infected fish could spread antibiotic resistance through the food chain, with serious implications for human health. (76).

Abundance profiles in the gut microenvironment are influenced by various interactions between species, which impact growth and loss rates (77). Think of bacterial communities as social networks, where members interact with each other in various ways, including competing for nutrients, cooperating through cross-feeding, communicating via secretion, and detecting substances outside their cells (78). Additionally, organisms can indirectly affect other community members by modifying their environment, a phenomenon known as “niche construction theory” (79). The gut microbiota might also be influenced by the presence of ecological factors, including biotic and abiotic stressors. Hence, to better understand how an existing microbial infection influences the functional properties of the gut bacteria, we investigated the hemolysin activity, biofilm formation, and host survival against the recovered bacterial isolates. Two important factors to consider when evaluating the potential virulence of a bacterial strain are its ability to produce hemolysin and form biofilms. These are the main criteria for classifying potentially pathogenic strains. We observed that the gut microbiota was infected with *P. penneri*, which had a higher number of pathogenic microbes, leading to the complete death of freshwater model fish species (*L. rohita*). The recovered bacterial isolates also exhibit significantly increased biofilm formation and hemolytic activity. Moreover, non-infected *L. rohita* showed more non-pathogenic isolates with lower hemolytic activity and biofilm formation. Mechanisms that can facilitate pathogen colonization include frequent disturbances (such as microbial infection) and evolutionary processes. When there are frequent disturbances, it can change indigenous communities by creating new niches. This can lead to new areas being invaded by foreign species or taken over by less dominant species (80). It appears that the ecological conditions and disturbances caused by bacterial infection promote the growth of pathogenic bacteria, while also inhibiting the growth of potentially beneficial and native species.

Interactions between infectious agents and their hosts occur in many different settings and levels. They can start at the single-cell level, spread through the host’s body, and even occur between hosts within a population, illustrating just how complex and dynamic these relationships are. That’s why it’s really important to explore the host-pathogen relationship from all these different angles. Doing so helps us better understand how infections happen and spread work (81). The immune system of fish is a complex network of innate and adaptive components that are present in all tissues. It plays a crucial role in defending the host against various external threats and internal disruptions of its natural balance. We employed a host-pathogen setup, where a virulent *P. penneri* strain was introduced into *L. rohita* as the challenge organism, and health status and immune responses were investigated. In the present study, the superoxide dismutase, catalase, and Glutathione peroxidase, which reflect the antioxidant response of animals, were significantly decreased, whereas the stress level, measured by cortisol activity, was significantly increased in challenged animals. In addition, the gene expression of *c3, hsp70, myd88, nod, il-6*, and *tlr22*, which are typically considered signs of an enhanced immune response or immune stimulation, was significantly upregulated. These immune genes, such as *IL-6*, play a crucial role in the initial inflammatory response by triggering a swift, robust reaction that leads to various inflammatory responses. Furthermore, they are key components of the immune system, participating in both adaptive and innate immune defenses, and have multiple functions, including direct killing, opsonization, regulation of the immune response, and mediation of inflammation (11, 38, 82). Therefore, increased pro-inflammatory cytokine expression might serve as a protective mechanism for fish to produce antiparasitic effects and restore homeostasis (83). Additionally, the increase in other immune genes in the treatment group suggests that the host’s defense system plays a vital role in its immunity and tolerance. Similar transcription profiles are reported in studies where researchers have found that bacterial infections modulate both innate and adaptive immunity gene expression (84, 85). Analysis of gene expression reveals that bacterial infections interact with the gut microbiome, influencing the host’s immune response and overall health.

In this study, we observed that expression of *myd8*8 in *P. penneri*-infected *L. rohita* was significantly upregulated (~280-fold). This might be primarily due to active host-pathogen interactions and the possible role of *myd88* in the innate and adaptive immune responses to neutralize the pathogenic *P. penneri* infection. Interestingly, *myd88* is reported to be involved in the innate immune mechanism and promotes antibacterial defense (86). *Myd88* plays a critical part in various signaling pathways in other fish, such as the Nile tilapia (87), as well as in *Labeo rohita*. Consequently, to confirm the correlation and gain insight into the molecular bases of the above-observed effects from the *in vivo* studies, we used molecular docking to assess the role of *myd88* protein in response to lipopolysaccharides (LPS) and O-antigen, which are responsible for bacterial virulence. Such *in silico* approaches are widely used today due to their precision and rapid results. The predicted structures were verified to estimate their accuracy and confirmed to be stable, reliable, and consistent. The structural organization of the rohu fish *tir* domain in the adaptor molecule *myd88* was previously observed to have low quality scores, in affinities and may thus provide a theoretical understanding of the proteins from *P. penneri*, serving as potential binding partners for *myd88*.

In conclusion, this study integrates comprehensive genomic analysis with detailed virulence characterization of *P. penneri*, including survival assays, histopathological evaluation, and hemolysin activity, alongside complete genome sequencing of the isolate. Our results demonstrate that *P. penneri* is a potentially pathogenic bacterium capable of altering gut microbial composition and phenotype, modulating host immune responses, and influencing disease susceptibility. Although bacterial infection contributes to the formation of distinct gut microbial communities, our findings emphasize the critical role of the host immune system in controlling infection through the induction of immune-related genes such as *c3, hsp70, myd88, nod, il-6*, and *tlr22*. Molecular docking analyses further suggest that *myd88* plays an important role in host–pathogen interactions by activating innate immune signaling pathways that may help prevent disease onset. Future studies focusing on gene expression dynamics and gut microbiota profiles in *P. penneri*-infected fish are needed to identify potential biomarkers for effective fish health management. In this regard, advanced approaches including metagenomics, metatranscriptomics, and single-cell analyses will provide deeper insights into host–pathogen interactions during *P. penneri* infection.

## Data Availability

’The datasets generated and/or analyzed during the current study are available in the NCBI repository, accession number OP554277. The names of the repository/repositories and accession number(s) can be found in the article/**Supplementary Material**.
