# Supplementary material for: Exploring the host-pathogen interaction and genome analysis of multidrug-resistant bacterial pathogen Proteus penneri isolated from Labeo rohita
Source: Front Immunol. 2026 Mar 13;17:1733414. doi: 10.3389/fimmu.2026.1733414 (PMC13021476; doi:10.3389/fimmu.2026.1733414)
Supplement: Supplementary file 1 [file DataSheet1.docx]

**Supplementary information**

**Interaction of *Myd88* protein with** **bacterial Lipopolysaccharides (lps) and O-antigen**

*Modeling of structures, validation of models and secondary structure prediction*

MYD88 sequence from L. rohita (NCBI Reference Sequence: XM_051098688.1) was retrieved. LrMYD88 shares 93.31% homology identity with [A0A672P1V7.1.A](https://swissmodel.expasy.org/repository/uniprot/A0A672P1V7?model=AF-A0A672P1V7-F1-model-v4) Myeloid differentiation primary response protein *Myd88*

AlphaFold DB model of *Sinocyclocheilus grahami* (Dianchi golden-line fish) (*Barbus grahami*), which is the highest homology among the experimental structures of *Myd88*.

The LPS assembly lipoprotein from *Proteus vulgaris* was retrieved from the NCBI database and similarly modelled. For the o-antigen, the template of [A0A198GD42.1.A](https://swissmodel.expasy.org/repository/uniprot/A0A198GD42?model=AF-A0A198GD42-F1-model-v4) O-antigen lipopolysaccharide chain length regulator

AlphaFold DB model of A0A198GD42_9GAMM (gene: A0A198GD42_9GAMM, organism: *Proteus myxofaciens* ATCC 19692) was used as it shared 81.45% of sequence identity. The above were used as the templates for homology structural construction using the software SWISS MODEL Protein-modelling Server (<http://swissmodel.expasy.org/>).

*Molecular docking analysis and protein-protein interaction analysis*

Active sites were thus considered for the protein-protein docking studies. Docking was carried out using both HDOCK and HADDOCK servers, providing both acceptable docking results. In HADDOCK, docking solutions are ranked using a clustering algorithm, with the best docking solution (having the lowest HADDOCK score and lowest Z-score) represented as the “most reliable cluster”. The HADDOCK score reflects the weighted sum of energy terms such as electrostatic, van der Waals, distance restraints (including AIRs), and many others–lower scores correspond to more reliable docking solutions. On the other hand, the Z-score is a measure of the spread of the HADDOCK scores, that is, the number of standard deviations the HADDOCK score of as given cluster is located from the average of all clusters-more negative Z-scores corresponds to more reliable docking solutions. Thus, these docked models were taken for further studies regarding protein-protein interaction.

**Supplementary Table S1** Water quality parameters in the experimental tanks

| **Water quality parameters** | **Values** |
| --- | --- |
| TAN (NH_4_^+^-N + NH_3_) (mg l^-1^) | 1 ± 0.22 |
| Nitrite (NO_2_^-^-N) (mg l^-1^) | 0.46 ± 0.04 |
| Nitrate (NO_3_^-^-N) (mg l^-1^) | 2.22 ± 0.12 |
| pH | 7.4 ± 0.12 |
| Dissolved Oxygen (mg l^-1^) | 6.91 ± 0.16 |
| Temperature (°C) | 28.7± 0.12 |

**Supplementary Table S1** List of primers used for amplification of bacterial 16S rRNA gene and host immune gene expression study.

| **Target gene** | **Primers** | **Sequence (5′-3′)** | **Annealing temperature (°C)** | **Amplicon size**  **(bp)** | **Reference** |
| --- | --- | --- | --- | --- | --- |
| 16S rRNA | UFF2 | GTTGATCATGGCTCAG | 52 | 1414 | Devi et al., 2022; Behera et al., 2018 |
|  | URF2 | GGTTCACTTGTTACGACTT |  |  |  |
| Heat shock protein 70 | *hsp 70*- F | GTGTTTGATGCCAGAGGCTG | 60 | 229 | Harikrishnan et al., 2020 |
|  | *hsp 70*- R | CTGCATTTGTCACCTTCTGCC |  |  |  |
| Glutathione peroxidase | *gpx*- F | GGGCTGGTTATTCTGGGC | 62 | 276 |  |
|  | *gpx*- R | AGGCGATGTCATTCCTGTTC |  |  |  |
| Superoxide dismutase | *sod*- F | GACAACACAAACGGCTGCAT | 60 | 170 |  |
|  | *sod*- R | CCTGACAGGGTCACCAGTTC |  |  |  |
| Catalase | *cat*- F | CGAGCATGTGGGAAAGACGA | 60 | 191 |  |
|  | *cat*- R | AGGACGGAAACAGAAACGCA |  |  |  |
| Toll like Receptor 22 | *tlr22*- F | CAGGTGGCGAGCTTCAGACT | 60 | 134 | Kole et al., 2017 |
|  | *tlr22*- R | CGGAGGTAGGTTCGTTTCTTCA |  |  |  |
| Complement Factor 3 alpha | *c3 alpha*- F | CCCTGGACAGCATTATCACTC | 60 | 155 | Ma et al., 2015 |
|  | *c3 alpha*- R | GATGGTCGCCTGTGTGGT |  |  |  |
| Myeloid differentiation primary response 88 | *myd88*- F | GCACATGCGTGTGGACCATC | 60 | 120 | Samanta et al., 2012 |
|  | *myd88*- R | GCAAAGACTGAGGGCAAACTT |  |  |  |
| Interleukin-6 | *il6*- F | GGACCGCTTTGAAACTCT | 54 | 212 | Robinson et al., 2012 |
|  | *il6*- R | GCTCCCTGTAACGCTTGT |  |  |  |
| Nucleotide-binding oligomerization domain | *nod*- F | CTGGTGGAGCAGGTGAAGAAC | 60 | 100 | Kole et al., 2017 |
|  | *nod*- R | TGCTGCTGCGCTGTATGATC |  |  |  |

**Supplementary Table S2:** Summary of the number of genes and databases where it has been found

|  | **Source** | **Genes** |
| --- | --- | --- |
|  | Victors | 1 |
| **Antibiotic Resistance** | CARD | 67 |
| **Antibiotic Resistance** | NDARO | 14 |
| **Antibiotic Resistance** | PATRIC | 149 |
| **Drug Target** | DrugBank | 85 |
| **Drug Target** | TTD | 11 |
| **Transporter** | TCDB | 257 |
| **Virulence Factor** | PATRIC_VF | 3 |

**Supplementary Table S3:** A summary of the AMR genes annotated in this genome and the corresponding AMR mechanism

| **Antimicrobial Resistance Genes** | |
| --- | --- |
| **AMR Mechanisms** | **Genes** |
| **Antibiotic inactivation enzyme** | ANT(6)-I, APH(3')-II/APH(3')-XV, APH(3')-III/APH(3')-IV/APH(3')-VI/APH(3')-VII, CatB family, OXA-50 family, PDC family |
| **Antibiotic target in susceptible species** | Alr, Ddl, dxr, EF-G, EF-Tu, folA, Dfr, folP, gyrA, gyrB, inhA, fabI, Iso-tRNA, kasA, MurA, rho, rpoB, rpoC, S10p, S12p |
| **Antibiotic target modifying enzyme** | Erm(B), RlmA(II) |
| **Antibiotic target protection protein** | Lsa(A), Tet(M) |
| **Antibiotic target replacement protein** | FabG, FabK, fabV, HtdX |
| **Efflux pump conferring antibiotic resistance** | EmrAB-OMF, EmrAB-TolC, MacA, MacB, MdtABC-OMF, MdtABC-TolC, MexAB-OprM, MexCD-OprJ, MexCD-OprJ system, MexEF-OprN, MexEF-OprN system, MexHI-OpmD, MexHI-OpmD system, MexJK-OprM/OpmH, MexPQ-OpmE, MexPQ-OpmE system, MexVW-OprM, MexXY-OMP, TolC/OpmH, TriABC-OpmH, YkkCD |
| **Gene conferring resistance via absence** | gidB |
| **Protein altering cell wall charge conferring antibiotic resistance** | GdpD, MprF, PgsA |
| **Protein modulating permeability to antibiotic** | OccD1/OprD, OccD2/OpdC, OccD3/OpdP, OccD4/OpdT, OccD5/OpdI, OccD6/OprQ, OccD7/OpdB, OccD8/OpdJ, OccK1/OpdK, OccK10/OpdN, OccK11/OpdR, OccK2/OpdF, OccK3/OpdO, OccK4/OpdL, OccK5/OpdH, OccK6/OpdQ, OccK7/OpdD, OccK8/OprE, OccK9/OpdG, OprB, OprB family, OprD family, OprF |
| **Regulator modulating expression of antibiotic resistance genes** | LiaF, LiaR, LiaS, OxyR |

**Supplementary Table S6** Bacterial isolates from gut samples of *L. rohita*

| **Treatments** | **Sampling time (h)** | **Bacterial species** | **Accession number** | **Family** | **Gram staining** | **Oxygen requirement for growth** |
| --- | --- | --- | --- | --- | --- | --- |
| **Control** | **48** | *Providencia alcalifaciens* | OR044752 | Morganellaceae | Gram-negative | Aerobic |
|  |  | *Proteus penneri* | OR044062 | Enterobacteriaceae | Gram-negative | Facultative anaerobic |
|  |  | *Citrobacter amalonticus* | OR044719 | Enterobacteriaceae | Gram negative | Facultative anaerobic |
|  |  | *Citrobacter amalonticus* | OR044721 | Enterobacteriaceae | Gram-negative | Facultative anaerobic |
|  |  | *Citrobacter amalonticus* | OR048059 | Enterobacteriaceae | Gram-negative | Facultative anaerobic |
|  |  | *Proteus terrae* | OR043646 | Enterobacteriaceae | Gram-negative | Facultative anaerobic |
|  |  | *Staphylococcus epidermidis* | OR039112 | Staphylococcaceae | Gram-positive | Aerobic |
|  |  | *Plesiomonas shigelloides* | OR039106 | Enterobacteriaceae | Gram-negative | Facultative anaerobic |
|  | **96** | *Providencia alcalifaciens* | OR044757 | Morganellaceae | Gram-negative | Aerobic |
|  |  | *Providencia alcalifaciens* | OR044935 | Morganellaceae | Gram-negative | Aerobic |
|  |  | *Providencia alcalifaciens* | OR047933 | Morganellaceae | Gram-negative | Aerobic |
|  |  | *Staphylococcus epidermidis* | OR039103 | Staphylococcaceae | Gram-positive | Aerobic |
|  |  | *Plesiomonas shigelloides* | OR054019 | Enterobacteriaceae | Gram-negative | Facultative anaerobic |
| **Treatment** | **48** | *Proteus penneri* | OR050833 | Enterobacteriaceae | Gram-negative | Facultative anaerobic |
|  |  | *Proteus penneri* | OR050834 | Enterobacteriaceae | Gram-negative | Facultative anaerobic |
|  |  | *Proteus penneri* | OR050835 | Enterobacteriaceae | Gram-negative | Facultative anaerobic |
|  |  | *Citrobacter amalonticus* | OR048075 | Enterobacteriaceae | Gram-negative | Facultative anaerobic |
|  |  | *Citrobacter amalonticus* | OR048076 | Enterobacteriaceae | Gram-negative | Facultative anaerobic |
|  |  | *Proteus terrae* | OR043671 | Enterobacteriaceae | Gram-negative | Facultative anaerobic |
|  | **96** | *Citrobacter amalonticus* | OR048074 | Enterobacteriaceae | Gram-negative | Facultative anaerobic |
|  |  | *Proteus penneri* | OR044714 | Enterobacteriaceae | Gram-negative | Facultative anaerobic |
|  |  | *Providencia alcalifaciens* | OR047930 | Morganellaceae | Gram-negative | Aerobic |
|  |  | *Proteus terrae* | OR043990 | Enterobacteriaceae | Gram-negative | Facultative anaerobic |
|  |  | *Proteus terrae* | OR044001 | Enterobacteriaceae | Gram-negative | Facultative anaerobic |

**Supplementary Table S5.** Multiple antibiotic resistance (MAR) indices and haemolysin assay of bacterial isolates recovered from gut samples of *L. rohita*

| S. No. | Treatment | Sampling time (h) | Bacterial species | **MAR indices of bacterial strain** | **Haemolysin assay of bacterial strain** | | |
| --- | --- | --- | --- | --- | --- | --- | --- |
|  |  |  |  | **MAR value** | **Clear zone diameter (mm)** | **Colony diameter (mm)** | **Ratio of clear zone and colony diameter** |
| 1 | **Control** | **48** | *Providencia alcalifaciens* | 0.19 | 0 | 6 | 0 |
| 2 |  |  | *Proteus penneri* | 0.11 | 27 | 6 | 4.50 |
| 3 |  |  | *Citrobacter amalonticus* | 0.11 | 0 | 5 | 0 |
| 4 |  |  | *Citrobacter amalonticus* | 0.11 | 0 | 6 | 0 |
| 5 |  |  | *Citrobacter amalonticus* | 0.11 | 0 | 7 | 0 |
| 6 |  |  | *Proteus terrae* | 0.11 | 0 | 6 | 0 |
| 7 |  |  | *Staphylococcus epidermidis* | 0.11 | 0 | 5 | 0 |
| 8 |  |  | *Plesiomonas shigelloides* | 0.15 | 29 | 6 | 4.83 |
| 9 |  | **96** | *Providencia alcalifaciens* | 0.15 | 22 | 7 | 3.14 |
| 10 |  |  | *Providencia alcalifaciens* | 0.11 | 0 | 6 | 0 |
| 11 |  |  | *Providencia alcalifaciens* | 0.11 | 0 | 7 | 0 |
| 12 |  |  | *Staphylococcus epidermidis* | 0.11 | 0 | 6 | 0 |
| 13 |  |  | *Plesiomonas shigelloides* | 0.11 | 0 | 5 | 0 |
| 14 | **Treatment** | **48** | *Proteus penneri* | 0.11 | 0 | 6 | 0 |
| 15 |  |  | *Proteus penneri* | 0.11 | 18 | 5 | 3.60 |
| 16 |  |  | *Proteus penneri* | 0.15 | 24 | 5 | 4.80 |
| 17 |  |  | *Citrobacter amalonticus* | 0.15 | 22 | 6 | 3.67 |
| 18 |  |  | *Citrobacter amalonticus* | 0.11 | 0 | 6 | 0 |
| 19 |  |  | *Proteus terrae* | 0.11 | 0 | 5 | 0 |
| 20 |  | **96** | *Citrobacter amalonticus* | 0.24 | 27 | 6 | 4.50 |
| 21 |  |  | *Proteus penneri* | 0.15 | 26 | 6 | 4.33 |
| 22 |  |  | *Providencia alcalifaciens* | 0.11 | 0 | 5 | 0 |
| 23 |  |  | *Proteus terrae* | 0.15 | 12 | 6 | 2.00 |
| 24 |  |  | *Proteus terrae* | 0.11 | 0 | 6 | 0 |

**Supplementary Table S6.** Zone of inhibition diameter (mm) by the bacterial strain recovered from fish gut samples

| **Treatment** | **Sampling time** | **Bacterial species** | **CFM** | **AMC** | **Ch** | **CPM** | **IPM** | **FO** | **CAZ** | **CIP** | **PB** | **PIT** | **Ka** | **NA** | **CL** | **TOB** | **NET** | **GEN** | **DIC** |
| --- | --- | --- | --- | --- | --- | --- | --- | --- | --- | --- | --- | --- | --- | --- | --- | --- | --- | --- | --- |
| **Control** | **48 h** | *P. alcalifaciens* | R | S | S | S | S | R | S | S | R | S | S | S | R | S | S | S | R |
|  |  | *P. penneri* | S | I | S | S | S | S | S | S | R | S | S | S | R | S | S | S | R |
|  |  | *C. amalonticus* | R | S | S | S | S | I | S | S | S | S | S | S | S | S | S | S | R |
|  |  | *C. amalonticus* | S | S | S | S | S | S | S | S | S | S | S | S | S | S | S | S | I |
|  |  | *C. amalonticus* | S | S | S | S | S | S | S | S | S | S | S | S | S | S | S | S | S |
|  |  | *P. terrae* | S | S | S | S | S | S | S | S | S | S | S | S | S | S | S | S | R |
|  |  | *S. epidermidis* | S | S | S | S | S | S | S | S | S | S | S | S | S | S | S | S | R |
|  |  | *P. shigelloides* | S | S | S | S | S | R | S | S | R | S | S | S | R | S | S | S | R |
|  | **96 h** | *P. alcalifaciens* | S | S | S | S | S | R | S | S | R | S | S | S | R | S | S | S | R |
|  |  | *P. alcalifaciens* | S | S | S | S | S | S | S | S | R | S | S | S | R | S | S | S | R |
|  |  | *P. alcalifaciens* | S | I | S | S | S | S | S | S | R | S | S | S | R | S | S | S | R |
|  |  | *S. epidermidis* | S | S | S | S | S | S | S | R | S | S | S | S | R | S | R | S | S |
|  |  | *P. shigelloides* | S | S | S | S | S | S | S | S | R | S | S | S | R | S | S | S | R |
| **Treatment** | **48 h** | *P. penneri* | S | S | S | S | S | S | S | S | S | S | S | S | S | S | S | S | R |
|  |  | *P. penneri* | S | S | S | S | S | I | S | S | R | S | S | S | R | S | S | S | R |
|  |  | *P. penneri* | S | S | S | S | S | R | S | S | R | S | S | S | R | S | S | S | R |
|  |  | *C. amalonticus* | S | S | S | S | S | R | S | S | R | S | S | S | R | S | S | S | R |
|  |  | *C. amalonticus* | S | S | S | S | S | S | S | S | S | S | S | S | S | S | S | S | R |
|  |  | *P. terrae* | R | S | I | S | S | R | I | S | S | S | S | S | I | S | S | S | R |
|  | **96 h** | *C. amalonticus* | R | I | S | R | S | R | R | S | I | S | S | S | R | S | S | S | R |
|  |  | *P. penneri* | S | S | S | S | S | R | S | S | R | S | S | S | R | S | S | S | R |
|  |  | *P. alcalifaciens* | S | S | S | S | S | I | S | S | S | S | S | I | S | S | S | S | I |
|  |  | *P. terrae* | S | S | S | S | S | R | S | S | R | S | S | S | R | S | S | S | R |
|  |  | *P. terrae* | S | S | S | S | S | S | S | S | R | S | S | S | R | S | S | S | R |

Following the guidelines of the Clinical and Laboratory Standards Institute (NCCLS, 2002; CLSI, 2015), susceptibility of recovered strains to different antibiotics is expressed as sensitive (S), intermediate (I) and resistant (R)

**Supplementary Table S7.** Survival assay of *Labeo rohita* challenged with different bacterial isolates recovered from fish gut samples.

| S. No. | Treatment | Sampling time (h) | Bacterial species | **Survival % (mean ± S.E.)** | | | | | | |
| --- | --- | --- | --- | --- | --- | --- | --- | --- | --- | --- |
|  |  |  |  | **24h** | **48h** | **72h** | **96h** | **120h** | **144h** | **168h** |
| 1 | Control | 48 | ***P. alcalifaciens*** | **20±3.3** | **0±0** | **0±0** | **0±0** | **0±0** | **0±0** | **0±0** |
| 2 |  |  | ***P. penneri*** | **50±3.5** | **30±4.8** | **0±0** | **0±0** | **0±0** | **0±0** | **0±0** |
| 3 |  |  | *C. amalonticus* | 100±0 | 100±0 | 100±0 | 100±0 | 100±0 | 100±0 | 100±0 |
| 4 |  |  | *C. amalonticus* | 100±0 | 100±0 | 100±0 | 100±0 | 100±0 | 100±0 | 100±0 |
| 5 |  |  | *C. amalonticus* | 100±0 | 100±0 | 100±0 | 100±0 | 100±0 | 100±0 | 100±0 |
| 6 |  |  | *P. terrae* | 100±0 | 100±0 | 100±0 | 100±0 | 100±0 | 100±0 | 100±0 |
| 7 |  |  | *S. epidermidis* | 100±0 | 100±0 | 100±0 | 100±0 | 100±0 | 100±0 | 100±0 |
| 8 |  |  | *P. shigelloides* | 100±0 | 100±0 | 100±0 | 100±0 | 100±0 | 100±0 | 100±0 |
| 9 |  | 96 | *P. alcalifaciens* | 100±0 | 100±0 | 100±0 | 100±0 | 100±0 | 100±0 | 100±0 |
| 10 |  |  | *P. alcalifaciens* | 100±0 | 100±0 | 100±0 | 100±0 | 100±0 | 100±0 | 100±0 |
| 11 |  |  | *P. alcalifaciens* | 100±0 | 100±0 | 100±0 | 100±0 | 100±0 | 100±0 | 100±0 |
| 12 |  |  | *S. epidermidis* | 100±0 | 100±0 | 100±0 | 100±0 | 80±4.1 | 80±0 | 80±0 |
| 13 |  |  | ***P. shigelloides*** | **50±5.8** | **50±0** | **40±2.5** | **40±0** | **40±0** | **30±3.1** | **30±0** |
| 14 | Treatment | 48 | ***P. penneri*** | **60±4.4** | **50±2.9** | **20±2.4** | **0±0** | **0±0** | **0±0** | **0±0** |
| 15 |  |  | ***P. penneri*** | **0±0** | **0±0** | **0±0** | **0±0** | **0±0** | **0±0** | **0±0** |
| 16 |  |  | ***P. penneri*** | **50±2.2** | **20±3.1** | **0±0** | **0±0** | **0±0** | **0±0** | **0±0** |
| 17 |  |  | ***C. amalonticus*** | **60±3.7** | **40±1.9** | **40±0** | **0±0** | **0±0** | **0±0** | **0±0** |
| 18 |  |  | *C. amalonticus* | 100±0 | 100±0 | 100±0 | 100±0 | 100±0 | 100±0 | 100±0 |
| 19 |  |  | ***P. terrae*** | **50±2.8** | **50±0** | **40±3.2** | **40±0** | **40±0** | **20±1.8** | **20±0** |
| 20 |  | 96 | ***C. amalonticus*** | **10±1.5** | **0±0** | **0±0** | **0±0** | **0±0** | **0±0** | **0±0** |
| 21 |  |  | ***P. penneri*** | **20±3.4** | **0±0** | **0±0** | **0±0** | **0±0** | **0±0** | **0±0** |
| 22 |  |  | ***P. alcalifaciens*** | **0±0** | **0±0** | **0±0** | **0±0** | **0±0** | **0±0** | **0±0** |
| 23 |  |  | ***P. terrae*** | **80±2.9** | **80±0** | **50±4.1** | **50±0** | **50±0** | **40±2.8** | **40±0** |
| 24 |  |  | *P. terrae* | 100±0 | 100±0 | 100±0 | 100±0 | 100±0 | 100±0 | 100±0 |

**Supplementary Table S8** Properties of the predicted models

| **Sl.No** | **Gene Name** | **ProSA Z-score** | **ERRAT score** | **Verify3D** |
| --- | --- | --- | --- | --- |
| 1. | *Myd88* | -8.3 | 93.02 | 61.62% |
| 2. | LPS | -4.3 | 100 | 40.11% |
| 3. | o-antigen | -5.82 | 99.69 | 41.13% |

**Supplementary Figure S1** Fish samples showing hemorrhage, ulcer and redness in fins and all over the body surface were collected from culture farms used for analysis


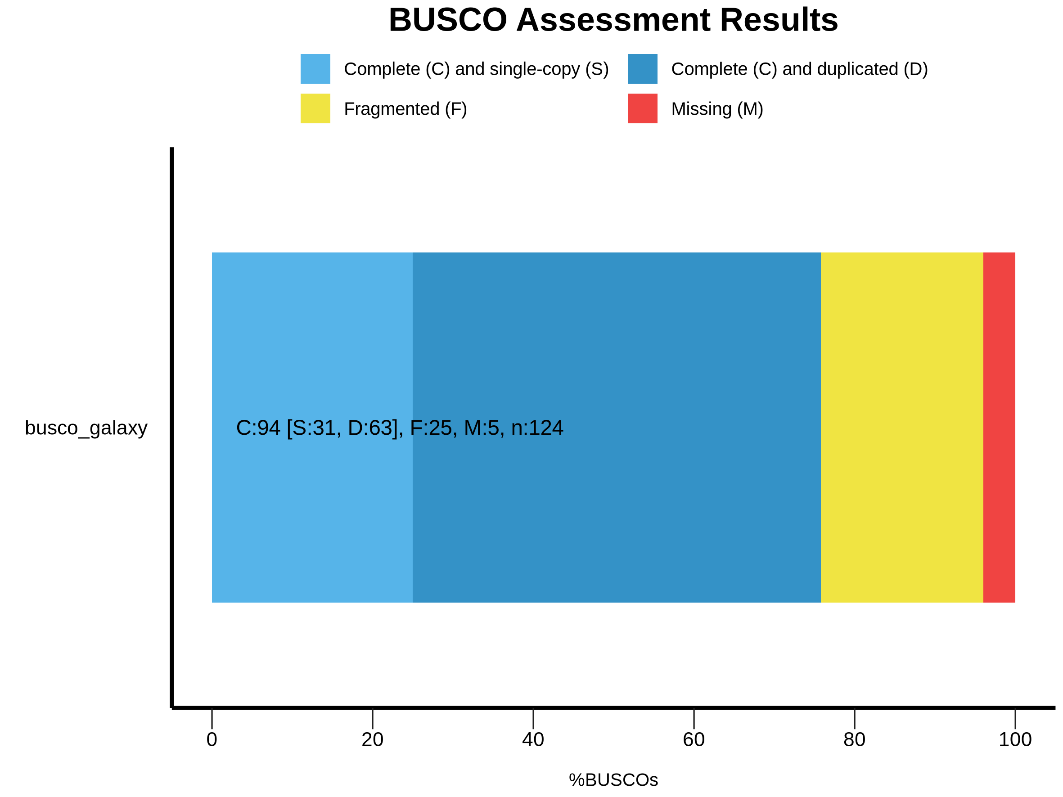
.

**Supplementary Figure S2. BUSCO Assessment for whole genome assembly of *Proteus penneri.***

**
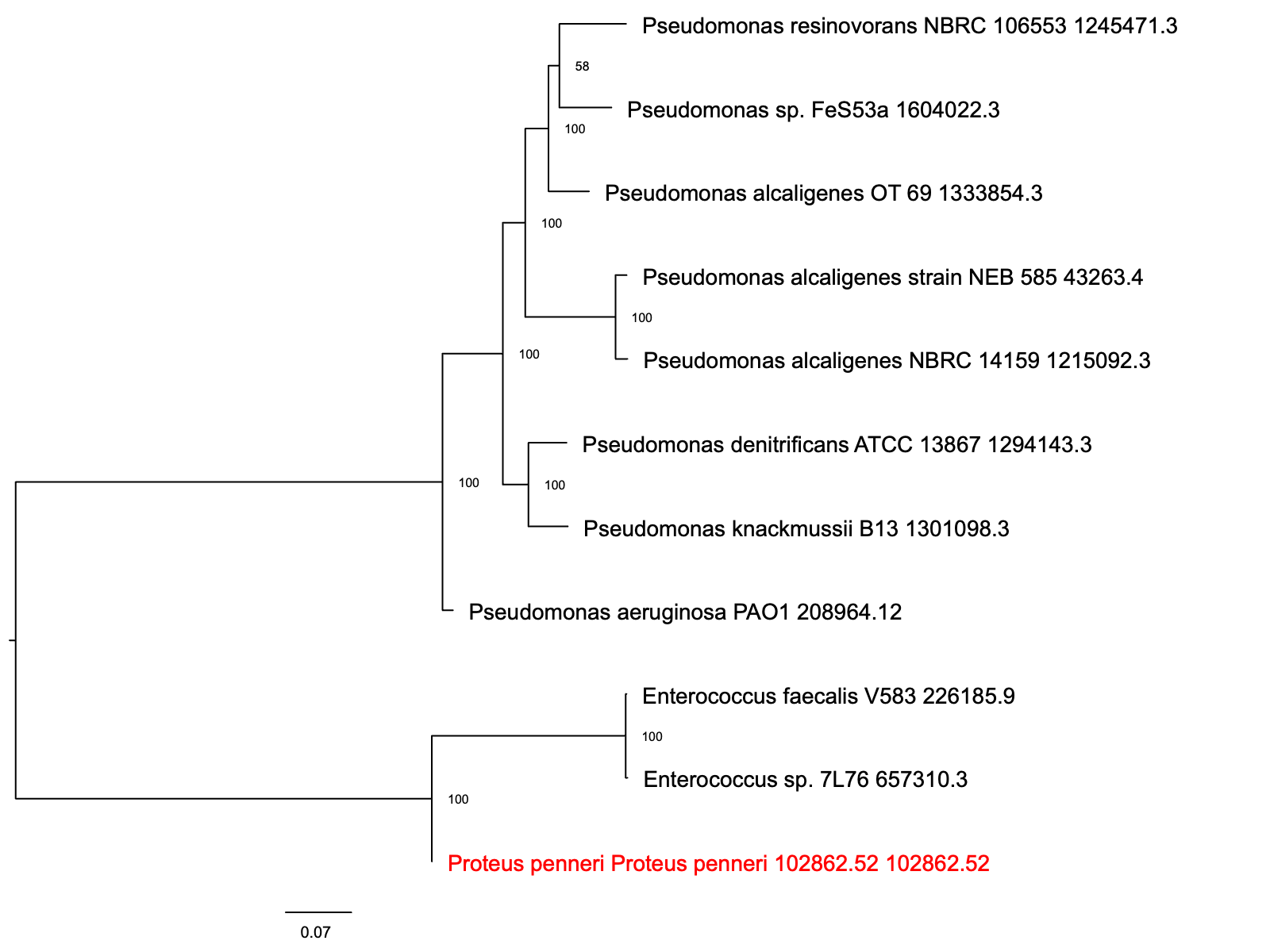
Supplementary Figure S3. Phylogenetic analysis of the reference genome of *Proteus penneri* along with representative genomes.**


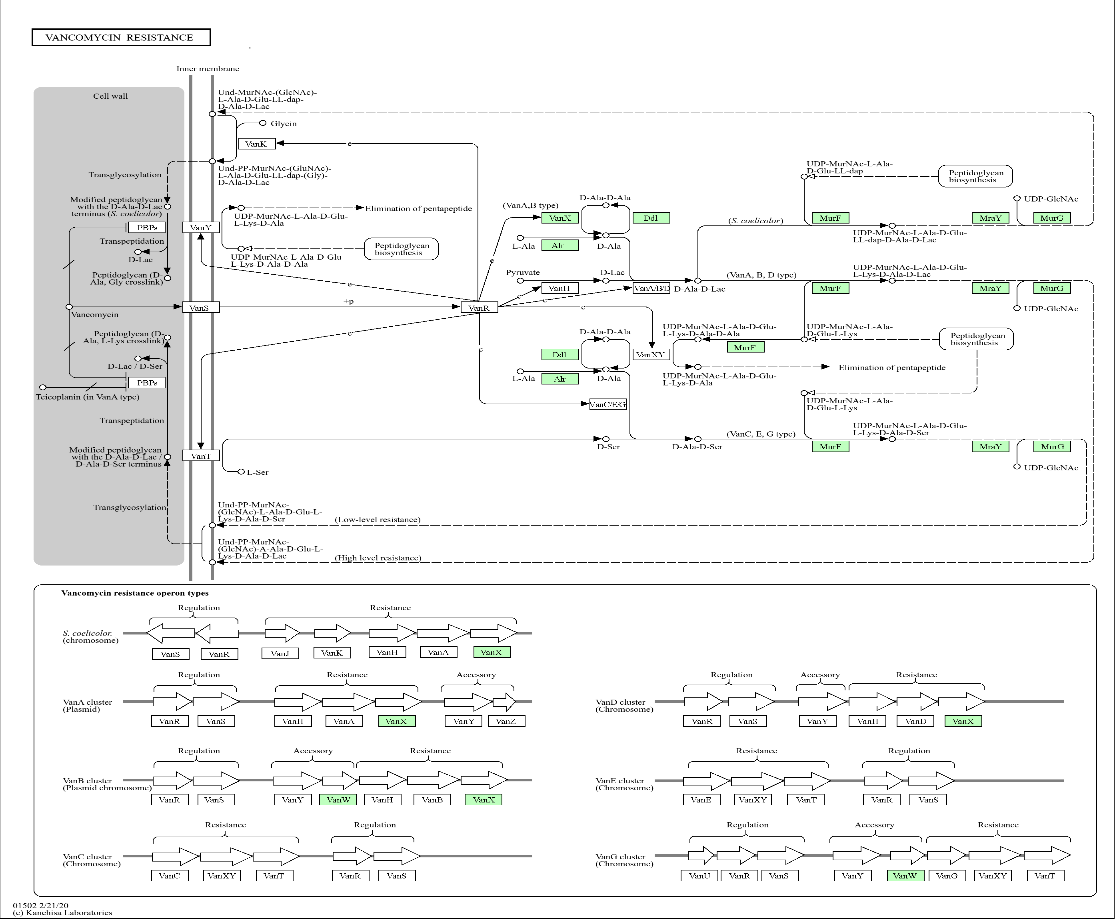

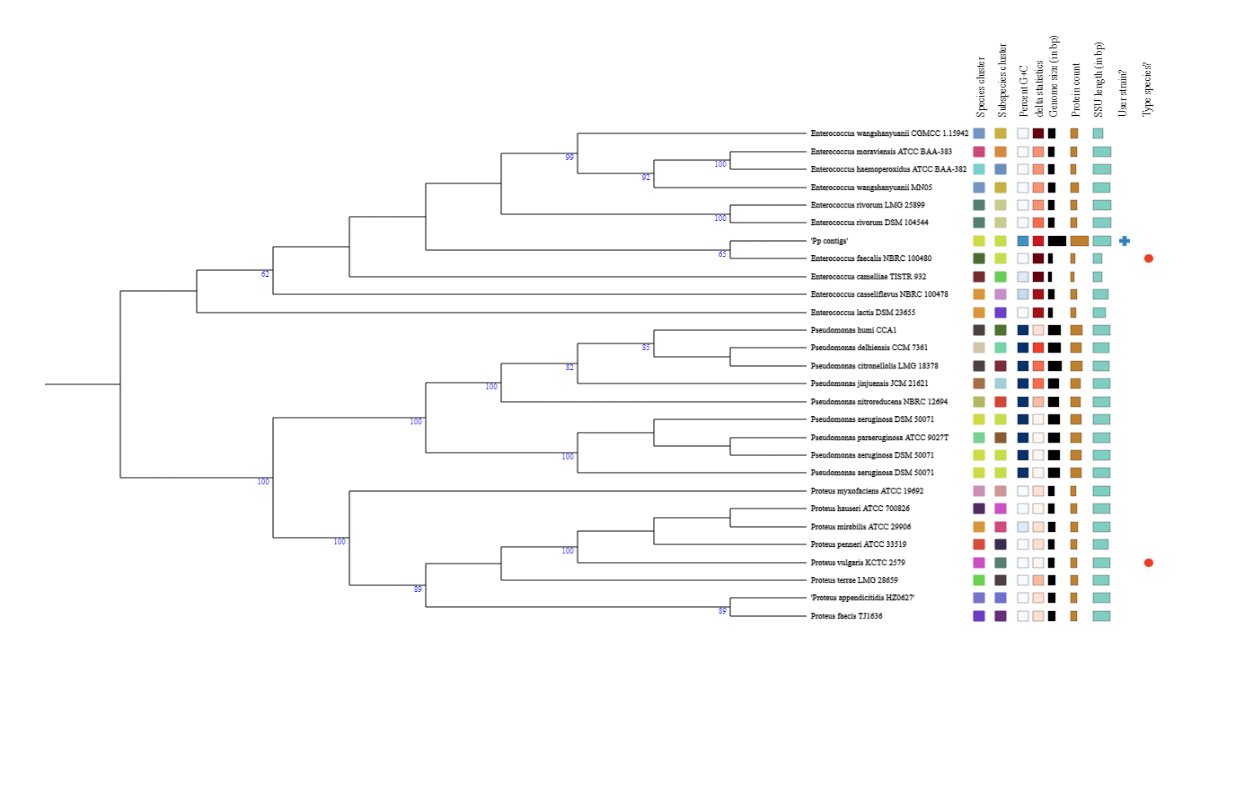

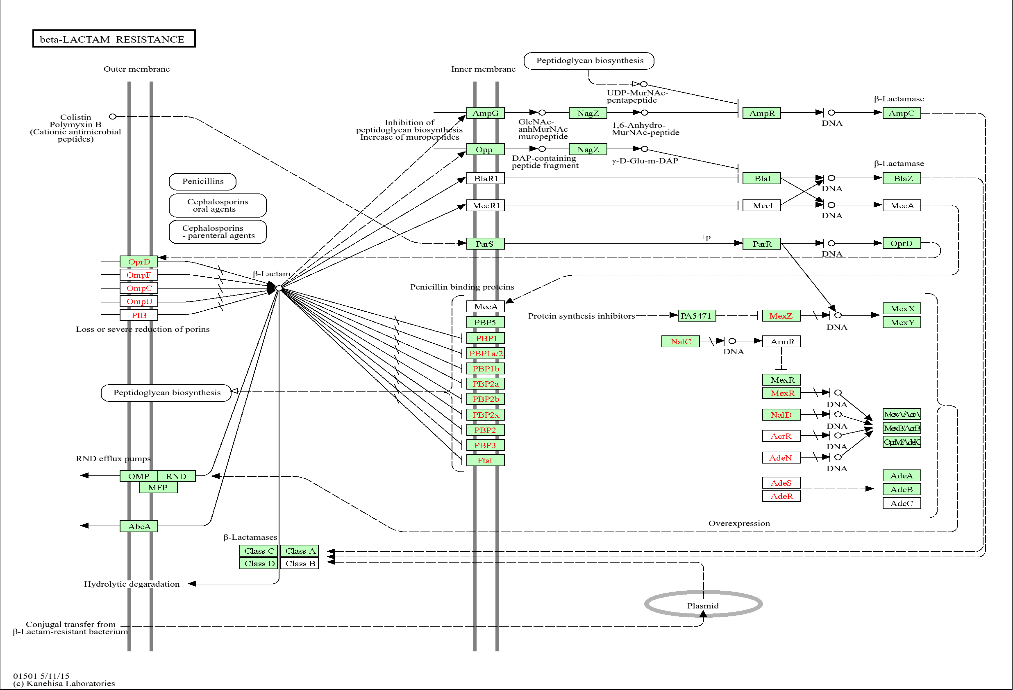

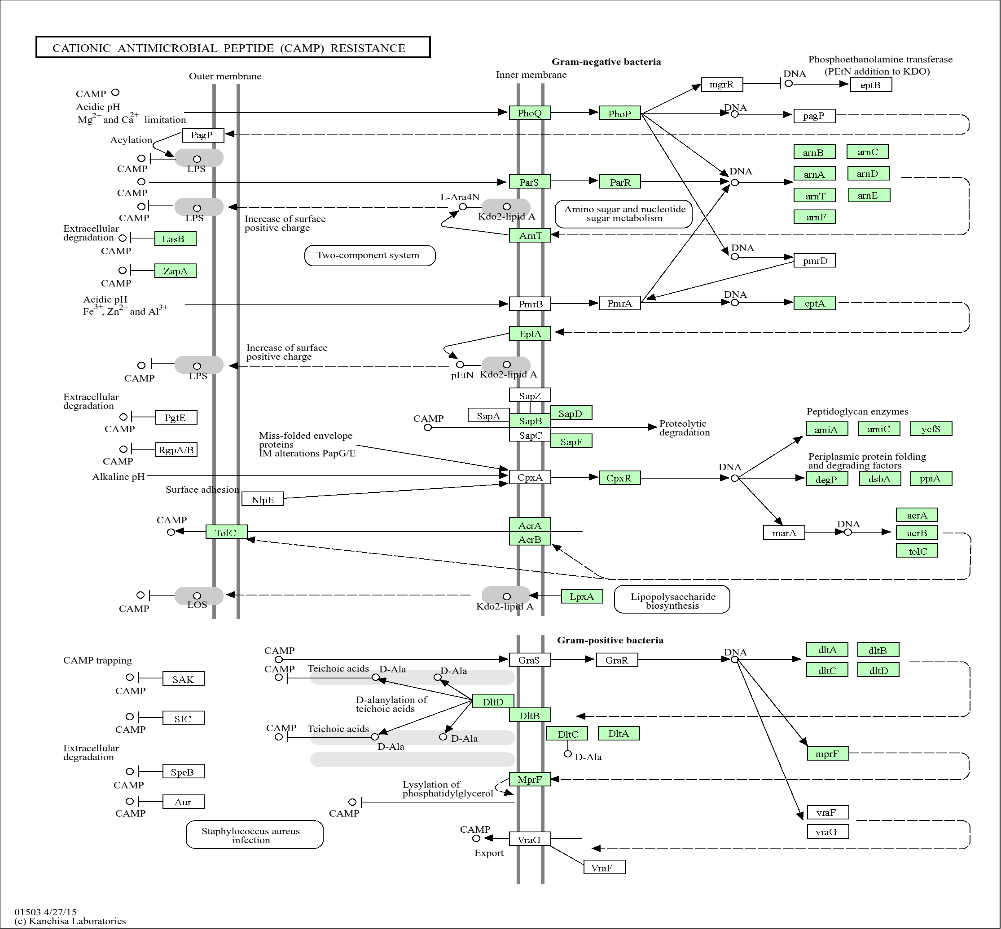


**C**

**D**

**A**

**B**

**A**

**Supplementary Figure S4. (A)** TYGS strain analysis of *Proteus penneri*. KEGG signalling pathway of **(B)** β-lactam resistance, **(C)** Vancomycin resistance and **(D)** CAMP resistance

**C**
